# Making sense of the cause of Crohn’s – a new look at an old disease

**DOI:** 10.12688/f1000research.9699.2

**Published:** 2016-11-16

**Authors:** Anthony W. Segal

**Affiliations:** 1 University College London, London, WC1E 6BT, UK

**Keywords:** Crohn’s, Inflammatory Bowel Disease, GWAS, Immunology, Infection, Bacteria, Gastroenteritis, Gene

## Abstract

The cause of Crohn’s disease (CD) has posed a conundrum for at least a century. A large body of work coupled with recent technological advances in genome research have at last started to provide some of the answers. Initially this review seeks to explain and to differentiate between bowel inflammation in the primary immunodeficiencies that generally lead to very early onset diffuse bowel inflammation in humans and in animal models, and the real syndrome of CD. In the latter, a trigger, almost certainly enteric infection by one of a multitude of organisms, allows the faeces access to the tissues, at which stage the response of individuals predisposed to CD is abnormal. Direct investigation of patients’ inflammatory response together with genome-wide association studies (GWAS) and DNA sequencing indicate that in CD the failure of acute inflammation and the clearance of bacteria from the tissues, and from within cells, is defective. The retained faecal products result in the characteristic chronic granulomatous inflammation and adaptive immune response. In this review I will examine the contemporary evidence that has led to this understanding, and look for explanations for the recent dramatic increase in the incidence of this disease.

## General Introduction

The enigma that is the cause of Crohn’s disease (CD) has puzzled clinicians and scientists from time immemorial. It is generally accepted that CD results from an aberrant immune response to commensal microflora in genetically susceptible individuals ^1^, however, the nature of the immune defects, the responsible microflora and the genetic susceptibility remain incompletely defined and actively debated. With advances in genomic technologies our understanding of this puzzling condition is evolving, and answers forthcoming. The purpose of this article is to undertake a holistic review of the aetiopathogenesis of CD in which historical concepts are integrated with recent discoveries.

### The bowel mucosa, an interface between faeces and the tissues

The distal ileum and colon contain >10 ^11^ bacteria per gram of faecal material ^2^, which pose an immediate threat to life if they penetrate into the underlying tissues. The bowel microflora are isolated by a thin film of mucus and a single layer of columnar epithelial cells with a surface area of approximately 32m ^2^, ^3^. The requirement for the absorption of fluids and nutrients by the bowel mucosa means that the bowel lining cannot simply be a tough impermeable barrier, and as a consequence provision must be made to defend the vulnerable mucosal epithelial cell layer against its contents. Mucus secreted by goblet cells forms a continuous, weak, viscoelastic gel, lining, 5–500 μm thick ^4^. In addition to acting as a physical barrier and lubricant, the mucus is the site of action of a variety of antimicrobial mechanisms including secretory IgA, antimicrobial enzymes and peptides ^5^ and H _2_O _2_ generated by the DUOX electron transport chain ^6^. Despite these barriers, the separation of the tissues from the gut microbiome is not absolute, and even in health the mucosa is constantly penetrated by relatively small numbers of enteric organisms and soluble microbial products that gain access into the tissues ^7– 10^. Scattered amongst the epithelial cells overlying lymph follicles are Microfold (M) cells ^11, 12^, a unique intestinal epithelial cell (IEC) subset that are highly specialized for the phagocytosis and transcytosis of gut lumen macromolecules, particulate antigens and pathogenic or commensal microorganisms, which they transfer across the epithelium to mucosal macrophages and dendritic cells. This slow, constant, transit is important for the development, priming and maintenance of a potent immune system in the submucosa ^13– 15^. The protective role of the bowel immune system must be combined with tolerance to ingested antigens and commensal organisms to maintain homeostasis in a healthy bowel.

### The immune system in the bowel

The bowel is the interface between a dense population of microbes and the immune system. Although an in depth review of the immune system in the bowel is well beyond the scope of this review, it is important to briefly cover this subject because defects in innate immunity are central to the development of CD whereas aberrant adaptive immunity causes bowel inflammation of a very different type, and a range of largely inaccurate animal models of CD.

***Adaptive immunity***. Most of what is known of classical adaptive immunology relates to the immune system of the bowel, but there are in addition some specialised features unique to the intestinal mucosa ^16– 18^.

The mucosae and exocrine glands harbour the largest activated B-cell system of the body, amounting to some 80–90% of all immunoglobulin (Ig)-producing cells in humans ^19^. The major product of these lymphocytes is polymeric (p)IgA (mainly dimers) with associated J chain. Both pIgA and pentameric IgM contain a binding site for the polymeric Ig receptor (pIgR), or secretory component (SC), which is a requirement for their active external transport through secretory epithelia into the overlying mucus ^19^.

M cells, and intestinal dendritic cells, that phagocytose bacteria interact with B and T cells in the Peyer’s patches, inducing B cells to produce IgA directed against intestinal bacteria ^20^. IgA+ B cells home to the intestinal lamina propria and secrete IgA that is transcytosed across the epithelium and deposited on the apical surface. The transcytosed IgAs bind to luminal bacteria, preventing microbial translocation across the epithelial barrier ^21, 22^.

After the initiation of the immune response by antigen processing and presentation to B and T cells in Peyer's patches, primed lymphocytes leave the mucosa via the thoracic duct. Finally they migrate back to the mucosa where they exert effector functions.

There has been considerable recent interest in IL-23 and IL-17 in relation to the aetiology of CD. IL-23 is secreted by macrophages and dendritic cells and transforms naïve T cells into Th (T-helper) 17 cells, and promotes their expansion and maintenance ^23^, and they then produce IL-17, IL-21 and IL-22 ^24^. IL-17 induces numerous cell types including T-cells, mast cells, macrophages, neutrophils, keratinocyte, and natural killer cells to produce a raft of pro-inflammatory mediators including IL-1b, IL-6, IL-8, IL-11, Gro-α, G-CSF, GM-CSF, IL-4, IL-5, IL-13, IgE, and eotaxin ^25^. An important outcome of this cytokine cascade appears to be the recruitment of neutrophils to inflammatory sites ^26^. Despite the apparent importance of IL-17 for intestinal barrier function ^27^ and for diverse pro-inflammatory activities, there must be considerable redundancy in the pro-inflammatory repertoire as defects in the IL-17 pathway are associated with a very narrow predisposition to disease in the form of mucocutaneous candidiasis ^28^. None of the hundreds of patients with this condition had CD ^29^.

IL-17 has been considered to be detrimental in CD as a consequence of its apparent pro-inflammatory actions. It is therefore of interest that a trial of the treatment of CD with monoclonal antibodies against IL-17 had to be stopped because of the deterioration of the patients’ condition ^30^.

Adaptive immunity in the bowel protects against commensal organisms, or those previously encountered in infections that were successfully overcome. This is accomplished by the production of a barrier of secreted IgA that permeates the lining mucus layer and by the production of specific IgG and IgM that opsonise penetrating organisms for phagocytosis. Immunity to pathogenic bacteria like *Salmonella, Shigella*, *Vibrio cholera* and *Escherichia coli* is generally not very potent or long lasting, which, together with the propensity of bacteria to mutate, makes vaccines relatively ineffective ^31, 32^. A degree of immunity does develop as a result of repeated reinfection in endemic areas but because this is not permanent, it is gradually lost after emigration to cleaner environments, which might be an important factor in relation to the subsequent triggering of CD by infection in individuals moving from regions of low to high prevalence of this condition.

***Innate immunity***. The submucosa of the bowel is particularly vulnerable to microbial invasion if the mucosal barrier is breached as large numbers of organisms can achieve rapid access and the conditions are conducive to microbial proliferation. There is inadequate time for adaptive immunity to take effect and reliance must be placed on the innate system to contain and eliminate potentially harmful stimuli. At its heart this means the rapid and florid release of pro-inflammatory cytokines from lamina propria macrophages ^33^, recruited from blood monocytes ^34^, mast cells ^35, 36^, eosinophils and innate lymphoid cells ^37– 39^ when activated by bowel contents. Paneth cells are specialised intraepithelial secretory epithelium of the small intestine that reside in small clusters at the base of crypts of Lieberkühn in the small intestine. Large secretory granules in these cells contain a wide variety of proteins, the most abundant of which are antimicrobials such as the alpha defensins that are discharged into the crypt lumen. These effector molecules also diffuse from the crypt and disseminate into the mucous layer that overlies the mucosal epithelium, where they contribute to the mucosal antimicrobial barrier ^40^.

Pro-inflammatory cytokines induce changes in the microvasculature ^41, 42^ leading to the extravasation of plasma proteins and to the recruitment of neutrophils ^43^. A critical concentration of neutrophils is required to eliminate invading bacteria ^44^ and immediately after bacterial penetration of the mucosa there is direct competition between bacterial replication and neutrophil recruitment and bacterial phagocytosis and killing. In the absence of specific antibodies, uptake of the foreign material is enhanced by non-specific opsonins like pentraxins, collectins and complement ^45^. The neutrophils then undergo apoptosis or necrosis and the purulent collection is most probably discharged into the bowel lumen, with the residual debris being phagocytosed and cleared by macrophages ^46^.

### Bowel inflammation in very early onset Inflammatory Bowel Disease (IBD)

Bowel homeostasis requires an intact mucosal barrier, itself requiring the integrated function of many different cell types and molecules, and the largest collection of immunological cells in the body to present an integrated defence against the intestinal microbiome. It is therefore not surprising that defects in genes coding for proteins required for the integrity of this barrier, or for normal immune surveillance, manifest as mucosal inflammation.

**Figure 1.**
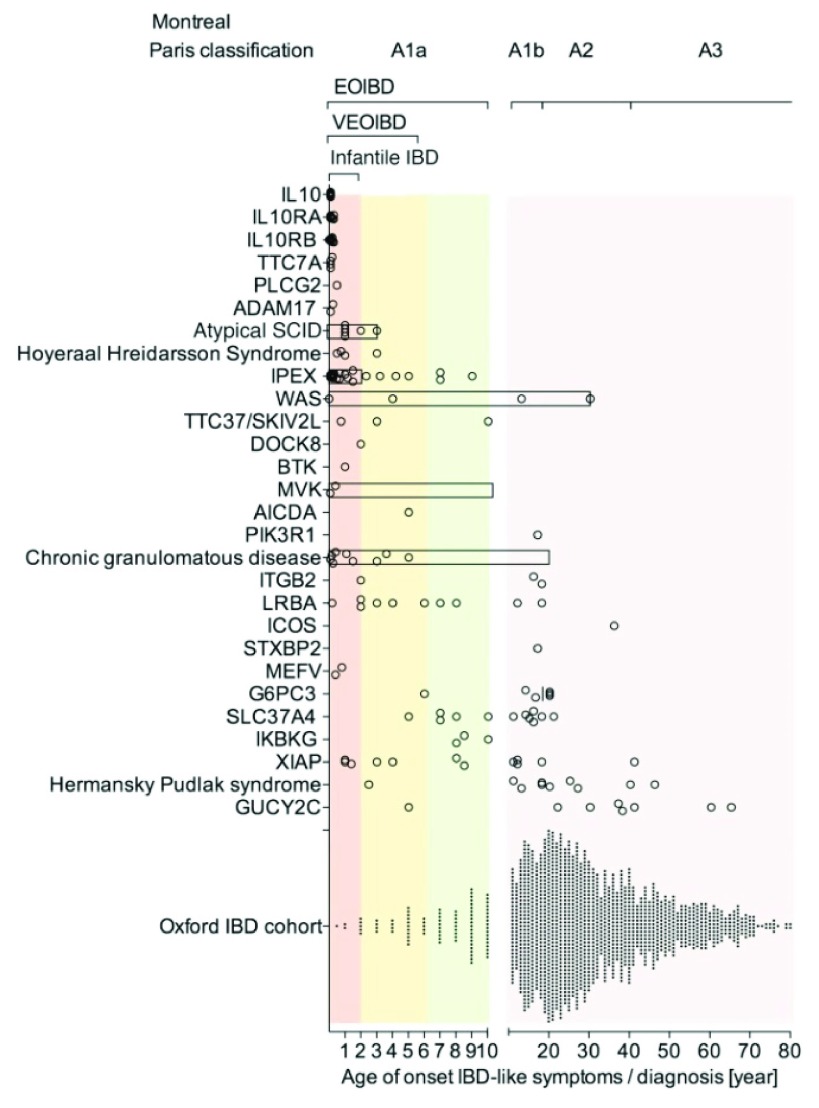
Age of onset of IBD-like symptoms in patients with monogenic diseases. Multiple genetic defects are summarized in the group of atypical Severe Combined Immunodeficiency (SCID), Hoyeraal–Hreidarsson syndrome, Chronic Granulomatous Disease (CGD), and Hermansky–Pudlak syndrome. By comparison, an unselected IBD population is presented (Oxford IBD cohort study; paediatric and adult referral-based IBD cohort, n = 1605 patients comprising CD, Ulcerative Colitis (UC), and IBD unclassified [IBDU]). Symbols represent individual patients. Bars represent the age range of case series if individual data were not available. Reproduced from 47 with permission from the publisher.

As might be expected, these conditions present very early in life, and because they affect the mucosa as a whole, they result in a diffuse, non-specific inflammation, predominantly in the large bowel where concentrations of bacteria are highest. Uhlig *et al.* ( Figure 1) ^47^ found that about 5% of their cases of IBD had infantile or very early onset disease. This will represent a much higher proportion of cases than that occurring in the general population, because most cases of IBD occur in adults and are handled in non-specialist facilities, whereas rare inherited diseases gravitate to specialist centres like those of Uhlig and his co-authors. The monogenic lesions identified provide important identifiers of the molecules required for bowel integrity and adaptive immunity.

**Figure 2.**
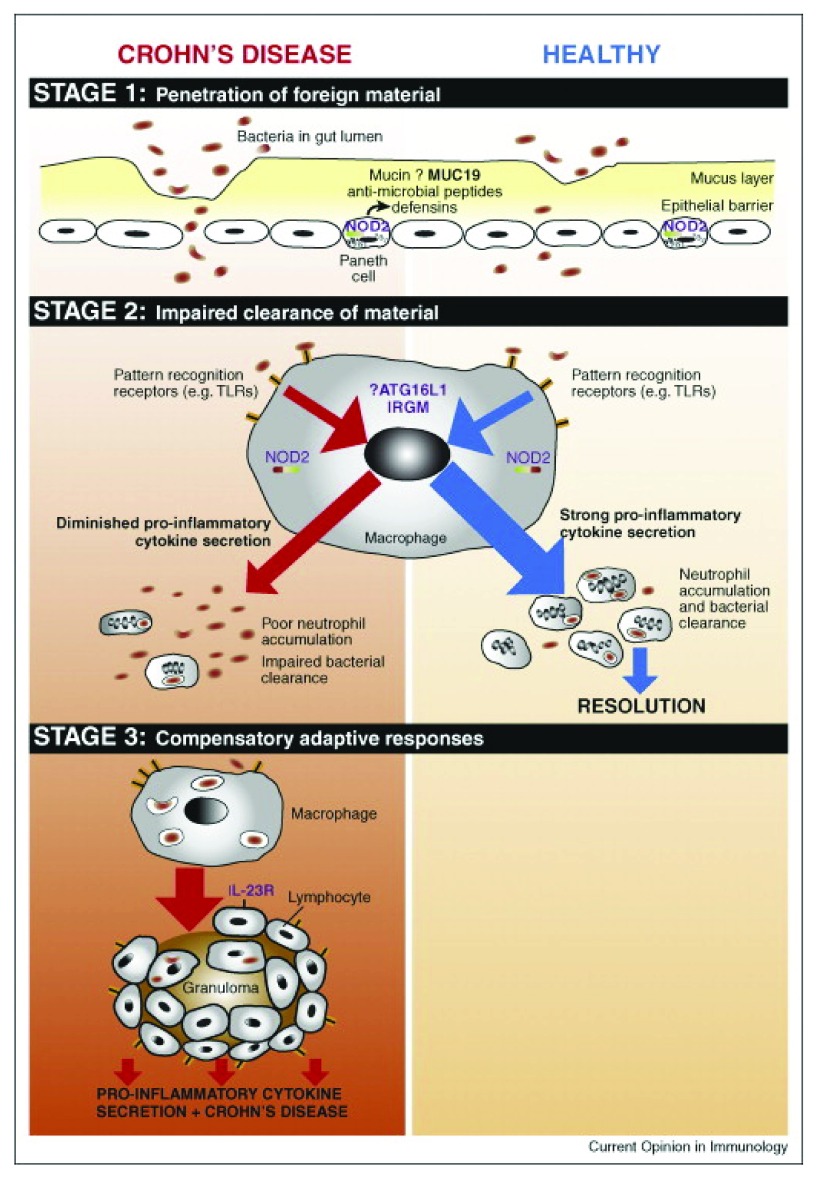
The immunopathogenesis of CD occurs in three temporally distinct stages. Penetration of luminal contents into underlying tissues occurs in stage 1, which may be facilitated by environmental factors such as infection, or inherent defects in the mucosal barrier. In healthy individuals, resident macrophages secrete pro-inflammatory cytokines in response to this material, resulting in neutrophil accumulation, clearance of the material, and thereby resolution. In CD patients, defective secretion of pro-inflammatory cytokines by macrophages results in impaired neutrophil influx and clearance of foreign material (stage 2). Subsequently, chronic inflammatory responses (stage 3) will be triggered, giving rise to the characteristic features of the CD lesion. From 87 Figure 1 (reproduced with permission from the publisher).

**Figure 3.**
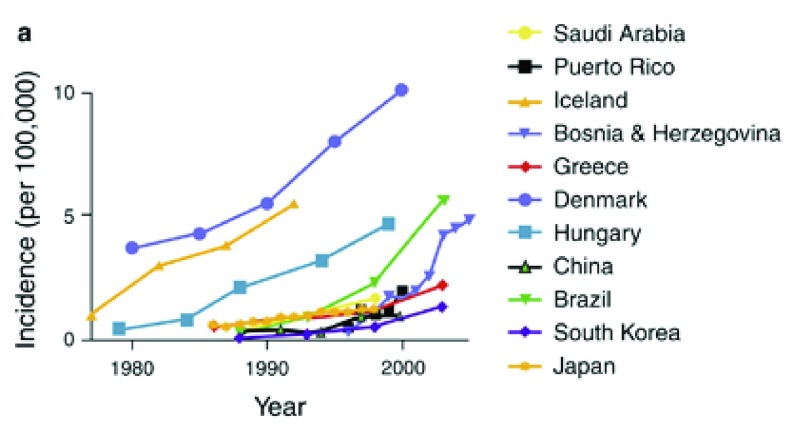
Increasing incidence of CD in several countries over time. Reproduced from 94 (with permission).

Mutations in the barrier function genes such as COL7A1, FERMT1, TTC7A and ADAM17 generally result in infantile bowel dysfunction and inflammation.

The severe immunodeficiency syndromes such as atypical Severe Combined Immunodeficiency (SCID) and Immunodysregulation Polyendocrinopathy Enteropathy X-linked Syndrome (IPEX) also generally have a very early onset and also do not have intestinal changes characteristic of either CD or UC. In contradistinction, the neutrophil defects, exemplified by Chronic Granulomatous Disease (CGD), and including Hermansky-Pudlak, congenital neutropenia and leukocyte adhesion deficiency all have a CD phenotype clinically, endoscopically and histopathologically, with a lot of perianal disease and granulomata evident on biopsy ^48^. The neutrophil defects generally present later than the abnormalities of mucosal barrier function, or the severe immunological diseases.

Mutations in the genes coding for IL-10 and IL-10 receptor both present very early in life, as seen in mucosal abnormalities and immunodeficiencies, and exhibit the bowel phenotype characteristic of defective neutrophil function. These observations would appear to be at variance with the prevailing view that IL-10 down-regulates macrophage function, and that the bowel inflammation in its absence is a manifestation of uncontrolled macrophage activation ^49^. IL-10 does appear to be required for normal intestinal development. Multisystem abnormalities were observed in the original description of the IL-10 knock-out mouse ^50^ in which there was a general enterocolitis with greatest abnormalities in the duodenum and jejunum, not locations associated with a high burden of commensal bacteria. Under specific-pathogen free (SPF) conditions the bowel lesions persisted, but were limited to the proximal colon. In addition the mice exhibited a severe growth defect, and were severely anaemic with a paucity of erythroid precursors in the bone marrow that was filled with myeloid precursors. The anaemia was unrelated to the extent of bowel involvement. These phenotypic features indicate that IL-10 is required for the normal growth and development of the bowel mucosa and haemopoetic tissue, in addition to its suppressant effect on macrophages ^51^.

The observed phenotype of patients with IL-10 and IL-10 receptor deficiency is in keeping with that of defective neutrophil function ^49, 52^. Almost all the patients showed evidence of bacterial infections in the form of folliculitis, and ear and respiratory tract infections. Most revealing was the almost universal occurrence of perianal disease with abscesses, fissures and fistulae that are highly characteristic of the neutrophil deficiency diseases like CGD ^53, 54^, Hermansky-Pudluck ^55^ and glycogen storage disease 1b ^56^. If the IL-10 deficient phenotype results from the impairment of normal cellular development and an immunodeficiency rather than an excessive, unregulated, macrophage response as proposed, then it might be expected that treatment with immunosuppressive therapy would be ineffective, which was indeed found to be the case ^52^.

It is also noteworthy that bowel inflammation is not a feature of the classical autoinflammatory diseases in which deregulated macrophage activation is a feature. These diseases include cryopyrin-associated periodic syndrome (CAPS) ^57^ in which activating mutations in the NLRP3 gene result in increased excretion of IL-1β excretion and other pro-inflammatory cytokines, and the haemophagocytic syndrome ^58^, in which the uncontrolled activation of antigen-presenting cells (macrophages and histiocytes) and T cells produces an exaggerated inflammatory response and cytokine storm.

### *Of mice and men -* mouse models of “IBD”

Mouse models of IBD have been extensively reviewed in the literature ^59– 61^. These models are very important because they are depended upon by clinicians and scientist trying to understand the causes of these diseases, and by the pharmaceutical industry attempting to produce drugs with which to treat them.

***Induced inflammation***. In general, impairment of the innate immune system as in mouse models of CGD or Wiscott-Aldrich does not result in the spontaneous development of bowel inflammation, although they do exhibit an exaggerated response to insult ^62^.

Predisposition to bowel inflammation may be exposed by reducing intestinal barrier function, thereby allowing access of the contained microbiome to the underlying tissues. Barrier function can be compromised through the genetic manipulation of proteins required for the production of mucus or the maintenance of epithelial integrity ^63^ or by the use of chemicals or infectious agents.

Chemical agents employed for this purpose fall into three main groups. Those that produce direct damage to the mucosa such as dextran sodium sulphate (DSS ^64^), acetic acid and carrageenan ^65^. The second group are those such as Haptens ( *e.g*. 2, 4-Dinitrochlorobenzene (DNCB ^66^) or Dinitrobenzene sulphonic acid (DNBS)) that induce an immune response. Finally infection with bacteria such as *Salmonella*, *E.coli* or *Citrobacter*, or parasites ^60^ may be utilised.

***Genetic models***. The advent of gene targeting technology has provided immunologists with powerful tools with which to explore the immune system. In the course of investigating its diverse components, hundreds of different genes have been knocked-out, some of which resulted in the spontaneous development of bowel inflammation. Because of this, these mice have been proposed as models of IBD.

Prominent examples of such mice include the IL-2 ^67^, T cell receptor (TCR)α/β ^68^, and IL-10 ^50^ knockout models. With the exception of IL-10-deficient mice, which possess some features of human CD, the majority of these models have diffuse colonic inflammation. A strain of mouse (TnfΔAREmice) was developed in which elements of the tumour necrosis factor (TNF) gene that are required to restrict the overproduction of this cytokine have been removed. In their absence the mice exhibit sustained over production of TNF which results in a diffuse arthritis and terminal ileal and caecal inflammation ^69^. IL-17 and IL-22 deficiency exacerbate induced colitis ^70^.

***Spontaneous models***. The C3H/HeJBir model of colitis was discovered by chance when mice in breeding colonies developed loose bowel actions ^71^. The pathology is characterised by spontaneous and chronic focal inflammation localised to the right colon and caecal region although not involving the small intestine. The colitis occurs in young mice and tends to resolve with age, without recurrence. The genetic mechanisms underlying these abnormalities remain to be identified.

SAMP1/Yit mice were developed from senescence-accelerated mice ^72^. They spontaneously developed ileitis and gastritis even under germ free conditions. The underlying aetiology is unknown but there is some evidence that the primary defect lies in the epithelial cell barrier and that B cells appear to play a role in the pathogenesis of inflammation at both sites ^73^.

***Adoptive transfer.*** One of the most commonly cited models for the study of the role of T lymphocytes in bowel inflammation in mice (as a proposed model of CD) is the adoptive transfer model in which T cells are transfused into SCID mice ^74^, resulting in bowel inflammation. By observing the effects of varying the populations of cells infused, conclusions have been drawn as to the regulatory interaction of the various cell populations. It is important to understand that these host SCID mice have hardly any B or T lymphocytes and, as their name suggests, are hypogammaglobulinaemic and severely immunocompromised. In the CD45RBhigh transfer model, first described by Morrissey ^75^ a subset of lymph node CD4 ^+^ T-cells, expressing high levels of the marker CD45RB (CD45RB ^hi^), were injected into SCID mice. The mice developed a wasting disease accompanied by massive hyperplasia of the intestinal mucosa with a dense infiltration of lymphocytes thought to be due to “an augmented, unregulated reaction towards higher levels of luminal-derived bacteria or bacterial products”. These changes were not seen when the animals were infused with unfractionated CD4 ^+^ or CD45RB ^lo^ cells, indicating that the extreme reaction to bacterial products ^76^ by the CD45RB ^Hi^ cells could be controlled by the CD45RB ^lo^ cells. Soon after, similar experiments were conducted by Powrie and colleagues ^77, 78^ who observed the same pathological changes in the bowel which they equated to those changes found in “inflammatory bowel disease” in humans. They showed that these changes could be prevented by antibodies to interferon-gamma and by recombinant IL-10 ^78^, and identified the cells in the CD45RB ^lo^ population responsible for controlling CD45RB ^hi^ induced inflammation as the population of suppressor T-cells called T-reg cells ^79^.

These observations and their extrapolation to human IBD sparked a large body of work into the role of regulatory T cells in the pathogenesis of IBD. Over the past decade, multiple groups have failed to find abnormalities in these cells in the intestines or blood of patients with IBD ^80^, not altogether surprising given the extreme artificiality of the animal model from which their presumed role in human disease was derived.

These mouse inflammation models are undoubtedly of great value in dissecting out immunological mechanisms and attributing roles to specific cellular populations and their associated cytokines. However, equating genetic mutations leading to bowel inflammation in mice with causal mechanisms of diseases in humans can have serious consequences as it may misdirect clinicians and scientists as to the underlying pathophysiology, and mislead pharmaceutical companies as to the relevant biological pathways against which to attempt to develop drugs. On the other hand, mouse models can be of great value when the problem is turned the other way around and they, and other animals like zebrafish, are used to validate the causality of molecular lesions found in association with disease in humans, for example those involving IL-10 ^61^ and ADAM17 ^81^.

## Classification of IBD

*“Medicine is learned by the bedside and not in the classroom. Let not your conceptions of disease come from words heard in the lecture room or read from the book. See, and then reason and compare and control. But see first.”*
*Sir William Osler*

IBD has referred to CD and UC because both can largely affect the colon and terminal ileum, however, although there may be overlap at the interface of these two conditions, their classical manifestations are quite different ^82, 83^. They are both syndromes, rather than specific diseases, where common clinical pictures are united by a common set of diagnostic criteria produced by similar pathophysiological mechanisms.

CD ^84^ usually involves the terminal ileum, and the caecum and colon to a variable extent, where the lesions are patchy, known as “skip lesions”, and associated with strictures, and fistulae between the bowel and other loops of bowel, the skin, and pelvic organs like the bladder and vagina. Outside the bowel, at the sites of transmural inflammation, the mesenteric adipocytes hypertrophy, covering the exterior of the bowel with a layer of protective fat, a process known as fat wrapping. Anal disease affects about 40% of these patients ^85^ exemplified by abscess, fistulation and skin tags. The inflammation is described as transmural, extending deep into the wall of the bowel, and contains diagnostic granulomata, collections of macrophages which represent a characteristic tissue response to retained foreign material. “The basic etiological factor in the case of all granulomas is probably the presence of a nidus of insoluble material which, if small enough is ingested by phagocytic cells, or, if too large, remains extracellular” ^86^. The central macrophages in these granulomata are surrounded by lymphocytes.

UC is very different in that it starts at the rectum and extends proximally, although occasionally, when it involves the whole large bowel there can also be involvement of the terminal ileum, a condition known as “backwash ileitis”. The inflammation in UC is superficial, being limited to the lamina propria, and the histological hallmarks are crypt abscesses and depletion of goblet cells that normally contain mucus.

Although a syndrome, the diagnostic features of classical CD are quite precise, and very different from the very rare cases of very early onset IBD, and the vast majority of genetically abnormal mice, both characterised by bowel inflammation rather than the clinical criteria used to diagnose CD or UC.

## The three phases of Crohn’s disease

A unifying model of CD pathogenesis has been proposed in which this condition develops in three temporally distinct phases ^87^:

• The trigger - gastrointestinal infection;
• A defective response to the consequences of this infection;
• A subsequent prolonged chronic inflammatory adaptive immune response.

### The trigger - Breeching the mucosa – The infectious environmental factor

***Epidemiology***. There is strong evidence for the role of an infectious environmental factor in the pathogenesis of CD. This is most obviously seen when populations or families emigrate from one country to another. A high proportion of family members have been documented as developing the disease after moving from Morocco to Belgium ^88^, from Albania to Greece ^89^ and from India to Canada ^90^. After being imported into the household enteric infections can spread to family members ^91^. At a population level, an increased incidence of CD has been described in recent immigrants from Ethiopia to Israel ^92^, and from Eastern European and Iraq to Sweden ^93^.

The epidemiology of CD has been the subject of a large body of work and multiple reviews. Most pertinent to this paper are issues concerning environmental influences, several of which are clearly associated with CD as outlined below.

***Temporal trends***. There has been a steep rise in the incidence of CD over the last few decades in economically advanced countries across Europe, North America and Australasia ^94– 96^. This is not purely an effect of increasing economic affluence because the incidence of CD is much lower in other economically advanced countries such as Japan and South Korea, although the incidence is now also rising in these countries ^97, 98^.

CD is generally more common in urban females of higher socioeconomic status ^96^, with a male to female ratio of about 1.5–2:1.

The difference in prevalence of CD by country could be partly explained by genetic factors; however, evidence from migration studies emphasise the importance of the environment. A limited number of studies investigating the incidence of CD among recent immigrants have been undertaken. The most informative of these assessed the risk of IBD in first- and second-generation immigrants to Sweden from many different countries ^93^. They found that overall risk of CD was lower in many groups of first-generation immigrants than in the native-born Swedish reference group but that in most groups of second-generation immigrants these decreased risks disappeared, and in some cases even exceeded those in the native Swedish population. First generation Middle Eastern immigrants to Australia developed CD at a much later age (∼57 years) than the second-generation who developed it at about 28 years of age, roughly the standard age in Western society ^99^. CD is very rare in Ethiopia but emerged in Ethiopian Jews migrating to Israel after a median lag of about 12 years after arrival ^92^. CD is also more common in Bangladeshi immigrants to England ^100^. Combined, these studies imply that immigrants from underdeveloped countries initially have a resistance to CD that wanes over the subsequent decade or so.

***Infection***. Infection has long been considered to cause CD. Attempts were made to transmit a CD agent from gut or lymph node tissue of patients to wild-type or immunodeficient mice ^101^. More granulomata were found in the mice receiving CD tissue, but that could have been due to the fact that the inflamed tissue contained enteric organisms or inflammatory cytokines. In the first description by Danziel in 1913 of what was later to be called Crohn’s disease, the similarity between “chronic interstitial enteritis” and Johne's disease in cattle ^102^, which is caused by infection with *Mycobacterium avium paratuberculosis*, was commented upon ^103^. Evidence that this agent was also responsible for human CD has been extensively sought ^104^ but has not been forthcoming ^105, 106^.

Several prospective studies have followed the course of patients after infections with enteric organisms and all have found an increased incidence of IBD as compared with uninfected control subjects ^107– 111^. In one of these ^110^ the risk was similar whether or not an infecting agent was identified, suggesting that it was the damage to the bowel rather than a specific infection that was important.

Enteric infections are most commonly caused by viruses, particularly *Norovirus* ^112^ and by *Campylobacter*, *Salmonellae*, *Shigella*, *Entamoeba histolytica*, *Cytomegalovirus* and *Yersinia* ^113, 114^. Particular attention has been paid to an adherent-invasive subgroup of *E. coli*, that has been linked to the development of CD ^115– 117^. The natural lesions produced by these organisms might provide some insight into those most likely to trigger CD. *Norovirus* mainly affects the proximal small intestine ^112, 118^ and Amoebic and Salmonella infections generally produce a diffuse colitis whereas the other infections result in lesions located in the terminal ileum and colon, with a patchy distribution, similar to those of the lesions of CD ^119– 126^.

**Figure 4.**
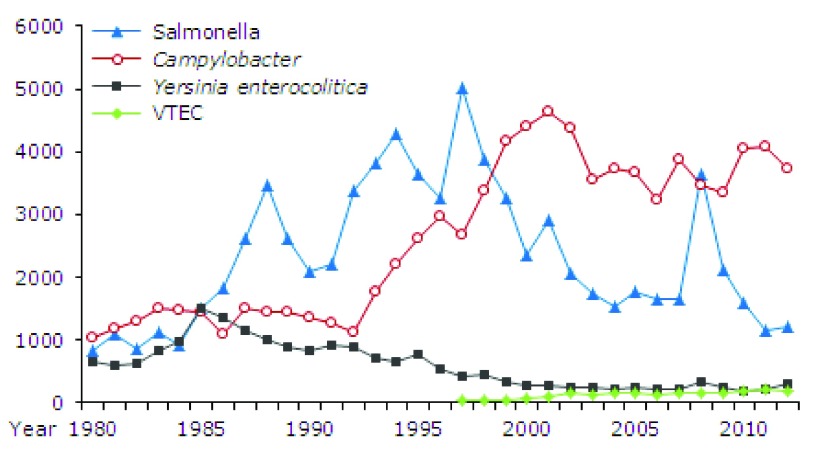
Number of recorded infections caused by *Salmonella, Campylobacter, Yersinia enterocolitica* and VTEC, 1980–2012. EPI-NEWS 12, 2013 from Statens Serum Institut, Denmark (reproduced with permission).

Whereas the incidence of most bacterial gastrointestinal infections is steady or falling, that induced by the commonest bacterial pathogen, *Campylobacter* is increasing in countries like North America, Europe, Scandinavia, Australia and New Zealand and Japan ^127^ ( Figure 4). This could be because this organism is a common contaminant of poultry, the consumption of which is increasing in these countries, however, broiler flocks are heavily contaminated with both *Campylobacter* and *Salmonellae* ^128^ and the incidence of infection by the latter is steady or falling ( Figure 4).

“Relapses” in cases of IBD have been reported to be associated with infections with various organisms including *Clostridium difficile*, *Shigella, Salmonella, Campylobacter, E. coli* and *Listeria* ^129^. These subsequent infections might be inducing the development of a novel set of Crohn’s lesions in a predisposed bowel, rather than recrudescence of the original disease, as a result of infection by different organisms. These subsequent infections could be predisposed to by the immunosuppressive treatments commonly used in this condition including corticosteroids, cytotoxic and biological agents.

Most gastrointestinal infections do not generally produce homogeneous mucosal damage but lead to focal areas of ulceration ^125, 126^, often in the ileocaecal region of the bowel. Because infection with invasive gastrointestinal pathogens is a stochastic process ^130^, the age at which this occurs is highly variable, as is the outcome after the infection, which will depend upon the severity of the infection, extent of ulceration, quantity of bowel contents gaining access to the tissues and to the effectiveness of the innate immune response.

***The microbiome, prebiotics, probiotics and faecal transplants.*** In the search for possible causal infectious agents, stool samples from CD patients have been extensively cultured and examined without a positive result (see for example 131). This is not entirely surprising because the average time from the onset of symptoms to diagnosis of CD is over six months ^132^ by which time an infectious organism will have been eliminated if it was a triggering agent rather than the cause of a chronic infection. With the advent of next generation 16S rRNA gene sequencing the phylogeny and taxonomy of samples from complex microbiomes can be determined without the need for them to be viable or culturable. Dysbiosis of the faecal microbiome is well recognised in CD ^133, 134^, with a decrease in the abundance and diversity of the *Firmicutes* phylum and an increased abundance of *Proteobacteria*, and alterations in the fungal composition ^135^. Differences were also found between the microbiotas of CD patients with ileal and with colonic disease ^136^. This could reflect an epiphenomenon secondary to the disease process. Major alterations in the microbiota are induced by diarrhoea ^137^, enteral nutrition ^138^, antibiotics ^139^, which most of these patients receive ^140^, and by iron therapy ^141^ which is often prescribed because these patients are generally anaemic. In general, gut and mouth microbiomes display universal dynamics, unlike microbial communities associated with certain skin sites that are probably shaped by differences in host environment ^142^.

Because CD predominantly occurs in those regions of the bowel with a high bacterial count, and given the differences in the microbiotas in CD described above, attempts have been made to alter the intestinal microbiota in the treatment of this condition. Prebiotics are typically non-digestible, fibre rich materials, which stimulate the growth or activity of advantageous bacteria that colonize the large bowel, whereas probiotics are live microorganisms that are directly administered by mouth. Neither prebiotics nor probiotics have been shown to be beneficial in CD ^143– 145^. An alternative means of directly altering the intestinal microbiota is by faecal microbiota transplantation, the transfer of faeces from a healthy donor, to restore the intestinal microbiota of a diseased individual. Whilst this is a logical treatment for *Clostridium difficile* infection, which generally develops in a colon depleted of its natural microbiome by antibiotics, it has not been found to be effective in the treatment of CD ^146, 147^.

***The Hygiene Hypothesis***. The considerable increase in the incidence of CD in developed countries in recent decades ^148^ has been attributed to immunological changes to alterations in the environment as outlined in the Hygiene Hypothesis ^149^. This hypothesis ^150^ was first described by Strachan in 1989 who stated that “over the past century declining family size, improvements in household amenities, and higher standards of personal cleanliness have reduced the opportunity for cross infection in young families. This may have resulted in more widespread clinical expression of atopic disease” ^151^. Subsequently modern living conditions have been held responsible for the increasing incidence of a variety of so called “auto-immune” diseases, including CD, which have been attributed to exposure a reduced load of microbes of decreased diversity. Certainly CD is less common in rural societies where there is exposure to animals, pets and soil, bedroom sharing is more common, and there is less access to hot water for ablutions ^99^.

According to this theory, standards of hygiene are lower in lower socioeconomic societies, leading to a greater abundance and variety of gastrointestinal pathogens. This would lead to a high incidence of gastrointestinal infections in infancy and childhood, resulting in death ^152^ or immunity ^153^. CD is very uncommon in underdeveloped societies in Asia ^154^, South America ^155^, China ^156^ and sub-Saharan Africa ^157^ and the increase in its incidence is closely associated with the improvement in income and living standards. Enteric infections are endemic in these developing societies in which diarrhoea is a major cause of death in children less than 5 years of age ^158– 160^. The population in underprivileged societies also host a large burden of gastrointestinal helminths ^161^ and the low incidence of CD recorded in developing countries has been attributed to the high rates of gastrointestinal infections with these organisms ^162^. Helminthic infection was found not to be protective against CD in Denmark ^163^ and the outcome of several trials of iatrogenic infection with helminths as therapy for CD are awaited, but current evidence does not suggest that they will be efficacious ^162, 164^.

Immunity to enteric organisms is transient ^165– 167^, and may be strain specific ^168^, and would be boosted by frequent reinfection in less advanced countries. This herd immunity would be lost over time after immigration to socially advanced, cleaner, societies, which would accord with the later age of onset of CD in first generation immigrants. One could envisage a situation in which the population of more socially advanced countries are living under increasingly hygienic conditions and are exposed to a less diverse repertoire of the enteric microorganisms capable of producing gastrointestinal infection. With less frequent gastrointestinal infection, the bowel is uninflamed, with less primed macrophages, mast cells and dendritic cells in the lamina propria and adaptive immunity is more restricted, and relatively feeble, through the lack of repeated boosts by infection, making the bowel vulnerable to attack by a novel or virulent organism.

If we postulate that the trigger for CD is enteric infection, how can the fact that the incidence of food-borne gastroenteritis is fairly steady in most developed countries ^169^ be reconciled with the rapidly increasing incidence of CD? Due to greater regulation and control of food production and distribution the incidence of foodborne outbreaks of disease have remained steady or have declined ^170– 172^.

It is important to consider the age distribution at which patients present with Crohn’s disease. It rises to a peak at between 20 and 30 years of age after which it demonstrates a steady decline, a pattern that is remarkably consistent, and very different from that of UC, across the geographical spectrum ^98, 173– 175^.

The peak incidence, generally at a later age than puberty, coincides with a stage in life accompanied by major lifestyle changes. These include the movement of individuals out of the family home, in which the ambient microbiome is likely to be relatively stable, into environments in which the risks of exposure to infection are much greater. The main two ways in which young adults are exposed to infectious enteric organisms is through the ingestion of contaminated food or fluids, or by person to person contact, the risk of both being increased by travel to places where exposure to novel organisms is more likely.

### Sexual transmission

**Figure 5.**
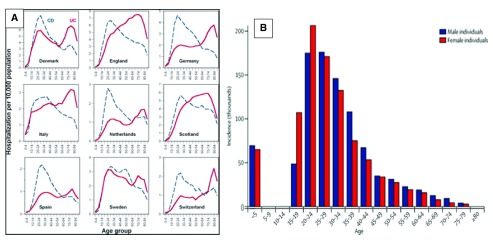
( **A**) The age distributions of Crohn's disease and ulcerative colitis in several European countries. (Reproduced from 173 with permission from the publisher). ( **B**) Global age-sex distribution of new HIV infections ^176^ (reproduced with permission).

Although enteric infections are generally considered to be foodborne, only about one half are in fact transmitted in this way ^177^, most of the rest being transferred by person to person contact. Sexual transmission is worthy of consideration as a means of transmission of faecal organisms between individuals because, as might be expected, the peak age for the acquisition of sexually transmitted diseases is very similar to that of CD ( Figure 5).

Epidemiological studies from developed countries have reported an increasing prevalence of invasive infections by *Entamoeba histolytica* ^178^, *Shigella* ^179^, *Cryptosporidia* ^180^ and *Campylobacter* ^181^, among men who have sex with men (MSM), which is not surprising because of the increased risk of exposure to coliform organisms by oral, anal and oro-anal sexual practises ^182^. The ingestion of as few as 10 virulent *Shigella* organisms can confer full-blown dysentery ^183^. It is easier to establish the causality of infectious outbreaks in these groups of individuals as compared with the general population, because they fall into more readily identifiable groupings which facilitate the epidemiological studies. It would be important to establish the incidence of CD in MSM, but diagnosis in these individuals is complicated by the relatively small proportions of individuals attending gastroenterology facilities, the presence of compounding factors such as “gay bowel” ^184^ and of sexually transmitted diseases like lymphogranuloma venerium ^185^ that can masquerade as CD.

**Figure 6.**
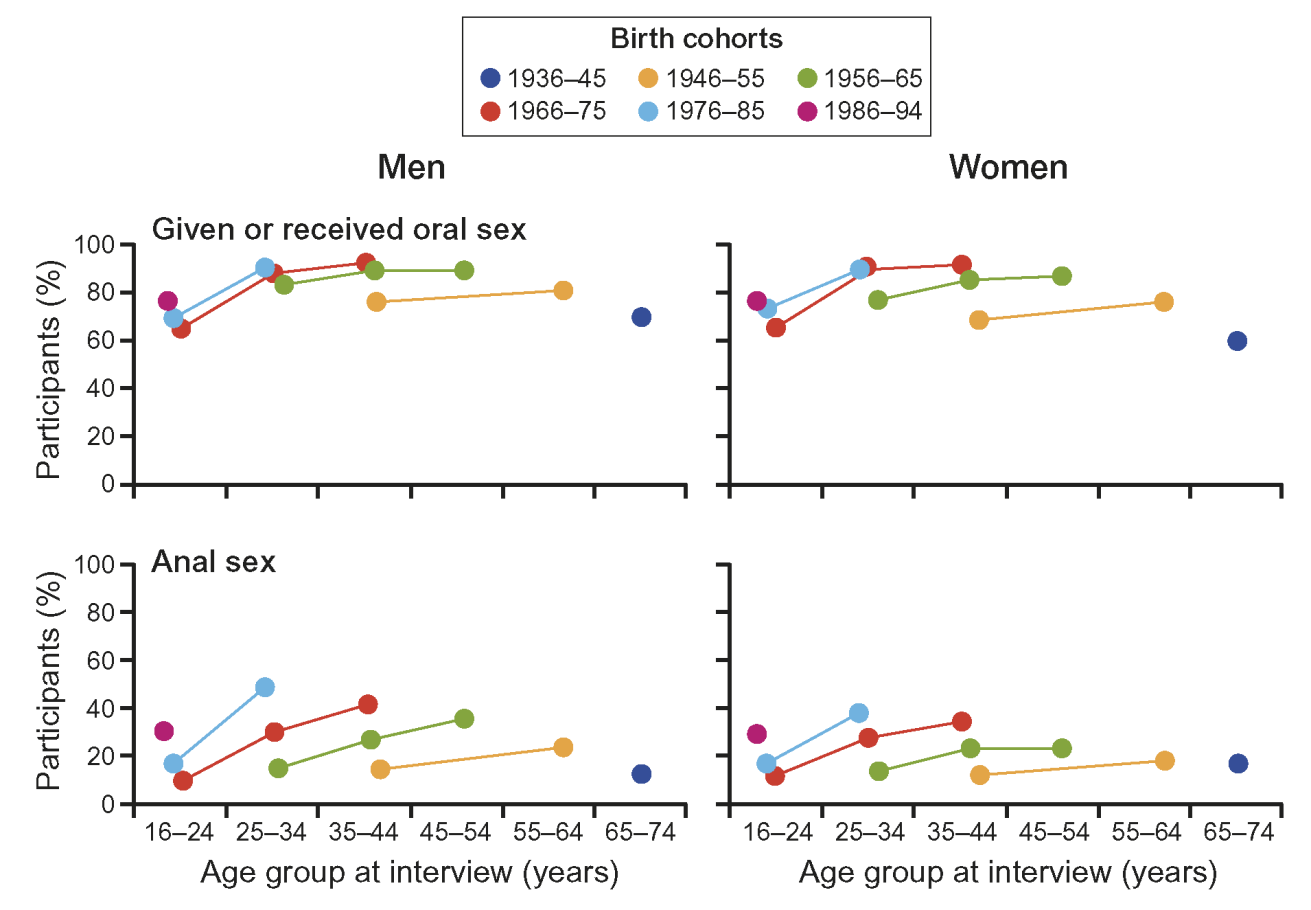
Heterosexual sexual practices in Britain. Redrawn from from the three National Surveys of Sexual Attitudes and Lifestyles ^190^. Each line connects values for the same birth cohort at different ages.

Given that oral and oro-anal sexual practises have been demonstrated to be responsible for the transmission of enteric infections in MSM, they must also pose a risk in other populations ^186, 187^. Although rectal bacterial flora are present on the perineum of both sexes ^188^ and in the vagina ^189^ it is unlikely that an increase in gastrointestinal infection would result from vaginal intercourse alone. Only about 5% of the sexually active individuals in countries like Britain ^190^ and the United States ^191^ are not heterosexual. In the heterosexual community the anal sex is practised by 30 – 40% of the population in England ( Figure 6) and North America, and fellatio and cunnilingus are almost universal ^190, 192^. In England the participation in anal sex has almost doubled over the last three decades, a similar increase to that of the incidence of CD. In terms of absolute numbers, approximately seven times more women than homosexual men engage in unprotected receptive anal intercourse ^193^. In addition, the ratio of homosexual to bisexual men is about 3:1, and the latter can act as “bridgers”, transmitting infections from men who have sex with men into the heterosexual community ^194^.

Those countries with a high standard of living and low rate of CD like Japan ^97^, Taiwan, China ^195^ Korea ^98^, Saudi Arabia ^196^ and Malaysia ^83^, appear to have low rates of heterosexual anal sex http://www.data360.org/pdf/20070416064139.Global Sex Survey.pdf ^197^ and in these countries the sex ratio of the disease, which is commoner in females than in males ^96^ in Western countries, is reversed, implying that men are particularly vulnerable to infection in these places. This does not appear to be due to a reporting bias because the sex ratio of UC in these countries matches that of Europe and North America.

Monogamous heterosexual couples develop complementary microbiomes ^198, 199^ which would suggest that the highest risk to infection of either partner through sexual contact would be in the early stages of a relationship, and that the risk to an individual would be related to the numbers of sexual partners, some of whom might be asymptomatic carriers of pathogenic organisms ^200– 202^.

Clearly the above arguments are conjectural and their validity will require validation through sound sociological, epidemiological and microbiological investigations.

Four other factors, smoking, antibiotics, appendectomy and invasive pneumococcal disease have positive correlations with the incidence of CD.

### Smoking

Smoking of tobacco is the strongest environmental influence on CD, roughly doubling the incidence ^203^ and relapse rate ^204^. Smoking and nicotine impair intestinal ^205, 206^ and gastric ^207^ mucosal blood flow. Adequate blood flow is central to the development of an effective acute inflammatory response. Smoking also reduces levels of acute inflammatory cytokines in the bowel wall ^208^ and lumen ^209^ in patients with CD.

### Antibiotics and appendectomy

There is an increased frequency of antibiotic use in CD prior to diagnosis ^96, 210^. The increased frequency of antibiotic use may be explained by an increased number of childhood bacterial infections. Similarly, an increased frequency of tonsillectomies ^210^ has been reported in CD and this may be an indication of recurrent pharyngitis. A further indication of a predisposition to infection in CD comes from the demonstration that these patients are more susceptible to invasive pneumococcal infection ^211^.

A history of a greater frequency of appendectomy in CD is also in keeping with an increased susceptibility to childhood infection in this condition ^96^.

### Phase two – a defective inflammatory response

*“Any infectious agent associated with Crohn’s disease is likely to be a widely distributed organism to which some people react abnormally - that is, the disease is unlikely to show the characteristic features of an infectious disease* ^212^ *.”*

As described above, there is very good reason to believe that the initiating lesion in CD is infection by one of a number of enteric pathogens. The key to comprehending how the pathological lesions of the disease then develop lies in understanding the response to that initial infection. The infection by the organisms described above is very unlikely to persist, or the causal connection would have been clearly established some time ago. This is also the reason that antibiotics are of only limited efficacy in the treatment of CD ^213^.

These patients are unlikely to be unduly susceptible to infection by these organisms, or else the onset would occur earlier, and systemic disease would be expected, as occurs with *Salmonellae* when the interferon-gamma/IL-12 axis is disrupted ^214^.

## Immunoparesis of the acute inflammatory response is the underlying Crohn’s phenotype

The underlying pathology in Crohn’s disease is the ineffective manner in which the faecal material entering the tissues through the damaged mucosa is dealt with. Infective damage to the mucosa followed by the entry of faecal material with a bacterial count of greater than 10 ^11^ bacteria per ml into the tissues poses an existential threat that must be dealt with vigorously. This is accomplished by the acute inflammatory response, a non-specific local reaction to tissue damage that recruits the innate immune system. It includes the secretion of inflammatory mediators from mast cells and macrophages, complement activation, markedly increased blood flow, capillary dilatation and permeability, the deposition of a fibrin network, and most importantly in the context of CD, a massive influx of neutrophil leukocytes, highly motile phagocytes that ingest and kill invading bacteria and fungi and digest foreign organic material.

The underlying, and unifying, predisposition to the development of CD is a systemic incompetence of this acute inflammatory response. I will deal with the evidence supporting this immunoparesis in some detail because these experiments have been performed on CD patients, and healthy control subjects, and in some cases patients with UC and represent a unique set of data that have not been repeated, possibly because of the invasive and uncomfortable investigations required to obtain them.

The delay in the recruitment of neutrophils to sites of trauma to the body by the innate immune response has been demonstrated in patients with CD in several different but complimentary ways. In 1976 I demonstrated that the accumulation of neutrophils in superficial abrasions on the arm called “skin windows”, was grossly deficient when compared with healthy subjects or patients with another chronic inflammatory condition, rheumatoid arthritis ^215^. It was observed that *“This abnormality of neutrophil function in Crohn's disease appears to be secondary to a defective acute inflammatory response as the neutrophils themselves were found to behave normally on in-vitro testing. A weak acute inflammatory response to particulate or antigenic material in the bowel wall could result in the chronic inflammation observed in this condition.”*

The next in these series of experiments was conducted on the ileal and rectal mucosa, and again on the skin ^216^. A small mucosal biopsy was taken from the ileum or rectum, and this was then followed 6 hours later by a further biopsy of the previous biopsy site, to determine the extent of the inflammatory response induced by the initial biopsy trauma. Once again there was a major delay in the recruitment of neutrophils in CD, and this was observed in both regions of the bowel. In addition to healthy subjects, control individuals with UC were studied and their neutrophil recruitment was normal. Trauma to the skin reproduced the impaired neutrophil recruitment into skin windows, as well as reduced secretion of IL-8 and IL-1β from them.

The direct injection of heat killed *E.coli* into the subcutaneous tissues of the forearm of normal subjects was followed by profound rise in local blood flow. This was considerably impaired in CD, but not in UC. Blood flow is important in recruiting innate immune cells to sites of inflammation and this already paltry vascular response in CD would be further compromised by smoking tobacco ^217^.

**Figure 7.**
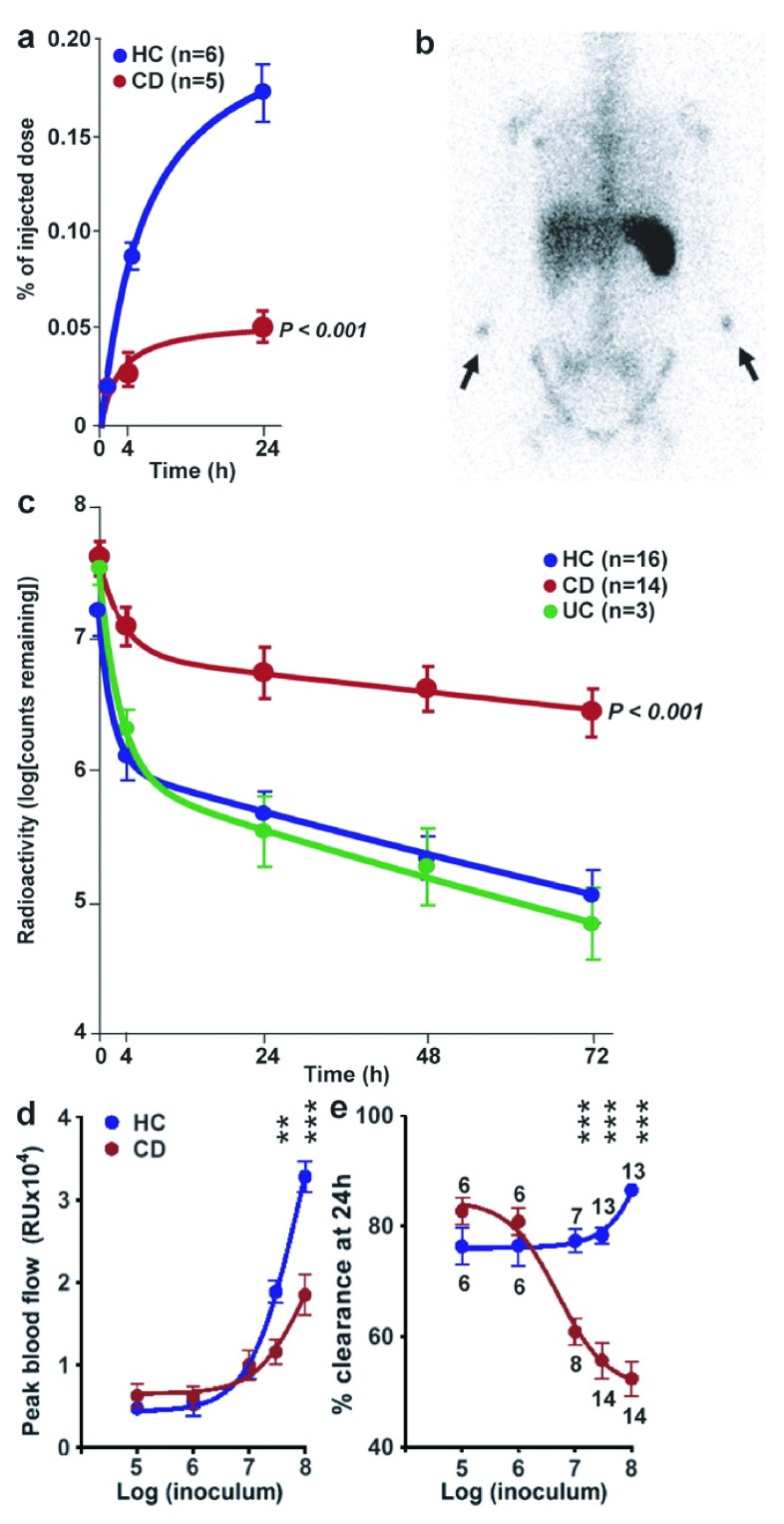
Neutrophil accumulation and subsequent clearance of *E. coli* from the tissues is markedly delayed in a dose-dependent manner in CD. Reproduced from 219 with permission from the publisher. ^111^Indium-labeled autologous neutrophils were injected intravenously at the same time as killed *E. coli* were injected subcutaneously into each forearm. ( **a**) Radioactivity measured over the injection sites showed a much smaller proportion of labelled cells accumulating in CD subjects. ( **b**) γ-Camera image of a CD patient at 24 h after injection, demonstrating focal accumulations of radioactivity at bacterial injection sites (arrows) and confirming lack of bowel inflammation. ( **c**) ^32^P-labeled killed *E. coli* were injected into the subcutaneous tissues of the forearm and radioactivity was measured at the skin surface. Clearance of radioactivity was much slower in CD than in HC or UC. Extrapolating these curves indicated that almost complete removal (99%) would take 10.2 and 7.1 d in HC and UC subjects, respectively, compared with 44.3 d in CD. ( **d** and **e**) Effect of increasing bacterial dose from 10 ^5^ to 10 ^8^ on blood flow ( **d**) and bacterial clearance ( **e**). The numbers of subjects studied in the dose response experiment are depicted in **e**. All results are expressed as mean ± SEM (**, P < 0.01; ***, P < 0.001).

The third of these experiments directly measured the accumulation of neutrophils at the site at which *E.coli* had been injected subcutaneously, and the rate of clearance of these organisms. In this study peripheral blood neutrophils were purified from the individual under investigation, labelled with the gamma-ray emitting radioisotope Indium-111 ^218^, and reinjected intravenously at the same time that unlabelled *E.coli* were injected subcutaneously into the forearms. The rate of accumulation of the radioactive neutrophils over the site of the injected bacteria was determined ^219^. A much smaller proportion of neutrophils were recruited to the injected bacteria in the CD subjects than in the HC or UC individuals ( Figure 7).

The next step was to radiolabel the *E.coli* with Phosphorus-32 and to then determine the rate of clearance of the bacteria from the tissues. This was a two phase process in HC and UC subjects with a very rapid initial clearance lasting about 4 hours followed by a slower phase, with total clearance being achieved by 7 to 10 days. In the CD subjects, initial clearance was much less efficient and total clearance was markedly delayed and was predicted to last from several weeks to infinity. This study showed unequivocally that coliform bacteria are cleared less efficiently from the tissues than normal in CD. It might be considered that this delayed recruitment of neutrophils to bacteria in the tissues should predispose these individuals to an increased incidence of clinically evident infections, which is not an obvious manifestation of CD. The reason for this apparent discrepancy is that the numbers of bacteria injected into the tissues were required to reach a certain critical load before the clearance defect was unmasked ( Figure 7e). In this study 10 ^6^ organism were cleared normally whereas 10 ^7^ were not, indicating that a significant bacterial load must enter the tissues before the clearance systems are overwhelmed. The bowel is the only location in the body where such a burden of microbes is readily available to enter the tissues.

### Phase three - The consequences of the failure to clear intestinal contents from within the bowel wall.

In the absence of an adequate acute inflammatory response and the complete clearance of the inciting agent by neutrophils, the retained foreign material produces a granulomatous inflammation ^220– 222^. *E.coli, Streptococci* and *Listeria* have been demonstrated immunochemically in macrophages, giant cells and lymph nodes of CD patients ^223^, and *E.coli* DNA has been identified in Crohn’s granulomata isolated by laser capture microdissection ^224^. The retention of this faecal material within the bowel leads to an intense adaptive immune response and the tissues become infiltrated with large numbers of T-cells. It is not therefore surprising that when actively inflamed CD tissues are biopsied, any number and variety of adaptive immune cells can be identified and immune mechanisms evoked in the pathogenesis of the condition. The macrophages and adaptive immune cells, reacting to the foreign antigenic material, will produce cytokines such as IL-1β and TNFα ^225, 226^ that lead to local inflammation and systemic symptoms ^227^.

The clinical picture of an inflamed bowel containing large numbers of macrophages and T-cells ^228, 229^ has led to the erroneous belief that Crohn’s was an autoimmune disease ^80^. It is however clear that the cytokines produced by these inflammatory foci in their response to foreign faecal material contribute to the local and systemic inflammation, and failure of mucosal healing, as evidenced by the, often dramatic, responses to anti-TNF drugs. However, only about half the patients respond to this treatment, and in those that do the response is often partial and temporary ^230^.

Chronic inflammation leads to fibrosis, or scarring ^231^, which in a hollow muscular organ causes narrowing, or stricture formation. Under some circumstances the material in the bowel wall undergoes liquefaction, as may occur with tuberculosis ^232^. This material then tracks to adjoining organs, possibly driven by the osmotic pressure produced by the breakdown of the organic material within the abscess, and discharges into them. This can then produce fistulae ^233^ between the these organs for example between bowel and bowel, bowel and skin, bladder or vagina. The perianal fistulae between the rectum and perineum are characteristic of CD and of immunodeficiencies of the innate immune system, particularly those of neutrophil function ^54^.

This failure to clear organic material from the tissues offers an explanation for the false positive Kveim tests observed in CD ^234^. The Kveim test ^235^ was designed to diagnose sarcoidosis, another chronic granulomatous disease. The intradermal injection of a crude homogenate of an extract of sarcoid tissue, usually from lymph node, produced epithelioid cell granulomas in subjects with sarcoidosis, reproducing those diagnostic of this disorder. Initially it was thought that the injected material contained some sarcoid specific factor, such as an infectious agent or antigen ^236^ but it has been recognised more recently that it relates to an abnormal host response:

“The "immune paradox" (delayed type hypersensitivity anergy in a setting of exuberant systemic granulomatous response) resists explanation. Its relationship to the Kveim test is poorly understood. Immunological investigations generated the thesis that the characterizing systemic granuloma arise as a fall-back reaction to inefficient cellular immune processing, due most often to impaired myeloid dendritic cell function of unknown cause” ^237^.

This is precisely the nature of the pathogenic mechanism in CD and it is therefore not surprising that positive tests are found in both conditions ^238^ and that both diseases occasionally coexist in the same individual ^239^.

## On the location of the CD lesions

Symptomatic lesions are largely confined to the terminal ileum, caecum and colon, probably due to the combination of mucosal damage by enteric infection coupled with the ready presence of massive numbers of bacteria to penetrate into the wall of the bowel when this happens. However, it is becoming apparent that the gastrointestinal tract is generally diffusely, sub-clinically, abnormal.

Oral manifestation of CD, particularly aphthous ulcers, are estimated to occur in 20–50% of patients ^240^. A prospective endoscopic study identified upper gastrointestinal (GI) manifestations of CD in 55% of 108 untreated, newly diagnosed adult patients with CD, irrespective of symptoms. All selected were free of *H. pylori*, infection with which, if anything, appears to protect against CD ^241^. About a quarter of the patients had lesions in both the stomach and duodenum and in about 20% they were in one or other of these organs. In roughly 2% of patients the gastric outlet is obstructed by a granulomatous inflammation requiring surgical intervention ^242^. Aphthous ulcers in the oesophagus were present in 7% of these subjects. Most of these lesions exhibited a granulomatous inflammation on histology.

In view of the systemic nature of the impairment of the innate immune system in CD, it is of great interest, although not altogether surprising that patients with CGD ^54^ exhibit very similar upper GI pathology. Aphthous ulceration and other oral lesions are common. Oesophageal, gastric and duodenal inflammation were detected in 21%, 74% and 37% of 78 patients ^53^. Large bowel lesions were present in the majority ^53^ and are indistinguishable from those of CD ^48, 243^. Between 4% ^244^ and 15% ^245^ of these patients also develop gastric outflow obstruction.

CGD is a condition in which there is a failure of microbial killing and digestion by neutrophils as a result of an absence of the respiratory burst produced by a NADPH oxidase, NOX2. Consequence, the pH of the phagocytic vacuole is too low for the efficient activity of the neutral protease digestive enzymes released into the vacuole from the cytoplasmic granules, and they fail to kill and digest the microbes ^246, 247^. The undigested material retained within the tissue is taken up by macrophages, producing the granulomata that give this condition its name.

Neutrophils play an important role in the debridement of wounds ^248, 249^, an important prelude and necessity for healing. It is possible that the upper GI inflammation that occurs in CD and CGD results from an impaired repair response to trauma and peptic digestion rather than infection in these locations.

## Identifying the molecular cause/s of the CD phenotype

### The abnormality of innate immunity in most cases of CD lies in the macrophages

**Figure 8.**
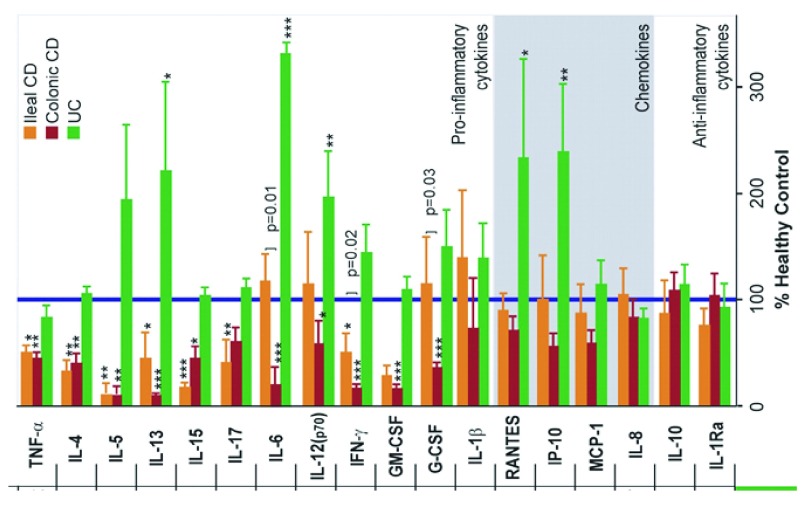
Proinflammatory cytokine secretion by macrophages from CD patients is deficient in response to *E. coli*. Cytokine and chemokine release expressed as a percentage of that secreted by HC cells (blue bar) from ileal and colonic CD patients (reproduced from 219 with permission from the publisher).

Defective secretion of pro-inflammatory cytokines in CD may be the explanation for the observed impairment in neutrophil recruitment ^219, 250– 253^. In CD, the neutrophils themselves are normal ^254^ and exhibit normal migration *in vitro* ^215, 255, 256^ and will migrate out of skin windows if chemoattractant substances are placed over them ^216^. In the absence of a primary abnormality of neutrophil function, CD macrophages showed defective secretion of pro-inflammatory cytokines, but normal release of chemokines, in response to stimulation with *E.coli* ^219^ ( Figure 8). The genes for these pro-inflammatory cytokines were transcribed and translated, but the proteins were misdirected to lysosomal degradation rather than secretion, suggestive of disordered vesicle trafficking.

The question then arises as to how anti-TNF drugs can be effective against a condition in which the secretion of TNF and other pro-inflammatory cytokines is impaired? The answer is in the timing of the different components of the immune system. The call to arms of the innate immune system is a very early and explosive secretion of pro-inflammatory mediators, including TNF. If the clearance of faecal material from the tissues is incomplete, it becomes walled off by macrophages, endotoxin diffuses into the circulation, and cells of the adaptive immune system are recruited ^257^. They secrete a wide array of mediators over the next weeks including TNF which acts as an amplifier of the response ^258^. It is of interest that in a recent study of high-resolution gene expression profiling using RNA sequencing of inflamed biopsies from patients with CD, UC and controls, levels of pro-inflammatory cytokines like TNF, IL-1β, IL-6 and IL-23 were all elevated to a lesser extent in CD than in UC ^259^. The very early secretion of TNF and other mediators is required to prevent the development of the Crohn’s lesions whereas at a later stage it is the TNF and associated mediators that produce the symptoms, which in some cases respond to anti-TNF treatment. This explains why anti-TNF therapeutics can both cause ^260^ and alleviate symptoms of the disease ^261^.

### What is the molecular cause of the impairment of acute inflammation?

There is a strong genetic component to the aetiology of CD. The sibling recurrence risk (risk of developing the disease in the context of an affected sibling) is approximately 13–36 ^262^ and approximately 12% ^263^ of CD patients have at least one affected first degree relative. Furthermore, the study of over 300 twin pairs has demonstrated a higher concordance of disease phenotype in monozygotic (30%) compared with dizygotic twins (4%) ^264^. While the twin studies support the role of genetic susceptibility, they also indicate the requirement for additional environmental or other factors for the development of overt disease. By far the most likely such factor is an enteric infection of sufficient severity to overwhelm the ability of the innate immune system to adequately clear the faecal debris from the bowel wall. However, this risk may be further modulated by additional environmental factors, such as smoking. This phenomenon, whereby a genetic predisposition to disease manifests in the presence of environmental precipitants is exemplified by alpha-1-antitrypsin deficiency, in which the predisposition to emphysema is exposed by smoking ^265^.

Technological advances have provided the means of interrogating the genetic basis of CD.

### 1. Linkage and Genome Wide Association studies (GWAS)

***Linkage.*** Linkage analysis (positional cloning) is a family based technique for identifying the possible location within the genome of causal mutations underlying genetic diseases ^266^. This is done by utilising markers of known location across the genome, such as microsatellites or single nucleotide polymorphisms (SNPs). The transmission of the markers through a family (or collection of families) is examined seeking those whose segregation closely follows the inheritance of the disease, thereby focussing attention on a small region (locus) in which the causal mutation might be found ^266^. Linkage analysis of affected sibling pairs with CD permitted the identification of a susceptibility locus on chromosome 16 (termed *IBD1*) in which mutations in the gene *NOD2* were subsequently identified ^267, 268^. *NOD2* mutations remain the most strongly associated common genetic variants associated with CD. Linkage is only a powerful tool when almost all cases of the disease in the families under study are caused by mutations in the same gene ( *i.e.* there is limited genetic heterogeneity) that are not seen in unaffected family members ( *i.e.* it is of high penetrance). Numerous factors can limit the effectiveness of linkage analysis such as: the presence of unaffected individuals that harbour the mutation (incomplete penetrance); individuals who develop the disease as a result of mutations in another gene or due to environmental factors (phenocopies); the requirement for the combined effect of two or more mutations (epistasis); or the requirement of the involvement of some environmental factor such as an infectious trigger (which will effectively reduce penetrance by not facilitating the manifestation of the underlying genetic predisposition in unexposed individuals). All of these factors are likely to have contributed to the limited success of linkage analysis in CD.

***GWAS.*** Genes reside on chromosomes which undergo recombination at meioses. Population level haplotypes arise due to the non-random positioning of crossing-over events. Haplotypes are characterised by a particular set of SNP genotypes. Depending on the ancestral origin and frequency with which a mutation has arisen in the population, it may occur on a particular haplotype and thus the SNP genotypes defining that haplotype will be enriched in patients harbouring the disease-causing mutation. Therefore, when comparing a large population of diseased individuals with healthy controls, SNPs tagging the underlying mutation should be enriched in the affected compared with unaffected individuals. In GWAS, a set of SNPs are genotyped in an attempt to cover the whole-genome and the above comparison made ^269^. One of the major problems with analysing many hundreds of thousands (or millions) of markers across the genome is that the large number of comparisons undertaken risks producing false positives. This necessitates the utilisation of a stringent p-value threshold for significance of p<5×10 ^-8^ ^270^. As a result very large sample sizes are required ^271, 272^.

One of the technical strengths of GWAS as an investigative approach is that DNA is easily obtained and once purified it is stable, enabling it to be conveniently stored and transported. Technological advancements have permitted high throughput SNP genotyping of large numbers of samples on an industrial scale. Furthermore, the GWAS approach has the advantage of being comprehensive (compared with candidate gene studies) and objective (at least up until the stage of data interpretation). There are however a number of limitations, for example incomplete genomic coverage. In addition, a major drawback is that the SNPs employed as markers must be relatively common in the general population, in order to give the study adequate statistical power, so this approach is typically unable to identify low frequency mutations, however penetrant or important.

When a SNP is found to be statistically significantly associated with a disease by GWAS, it can be because the polymorphism is itself pathogenic or, more commonly that it is tagging a closely located genetic variant whose genotype correlates with that of the tagging polymorphism (the two variants are in linkage disequilibrium). The precise location of the causal variant(s) underlying the association signal within the identified locus may be interrogated further by fine mapping (in which higher resolution association studies are conducted) or by resequencing the locus looking for plausible pathogenic variants such as coding variants or those that affect gene-expression (eQTLs).

Increasingly large GWAS have been performed on CD and the results meta-analysed ^273, 274^. No single, or small number, of penetrant mutations have been found that independently cause the disease. The latest study of over 20,500 CD cases and 41,600 controls of European ancestry identified 145 loci associated with CD at p<5×10 ^-8^. The mean OR of the top SNPs representing these 145 loci was 1.16 and the mean control allele frequency was 0.48. Four SNPs had an OR exceeding 1.5 of which three were within *NOD2* and the fourth was in *IL23R*. The mean difference in allele frequency between cases and controls was only 0.02 ^275^.

The very significant p-values obtained for the associated loci, led to the general perception that the molecular causes of CD have been identified. Individually the CD GWAS loci have very modest effect sizes ( *i.e.* a small difference in frequency in the control and CD populations), consistent with a polygenic model in which it is thought that that it is the combination of these minor influences that is causally important. However, in the latest meta-analysis, all 170 significantly associated loci combined account for only 10.9% of the disease “heritability” ^273^.

**Figure 9.**
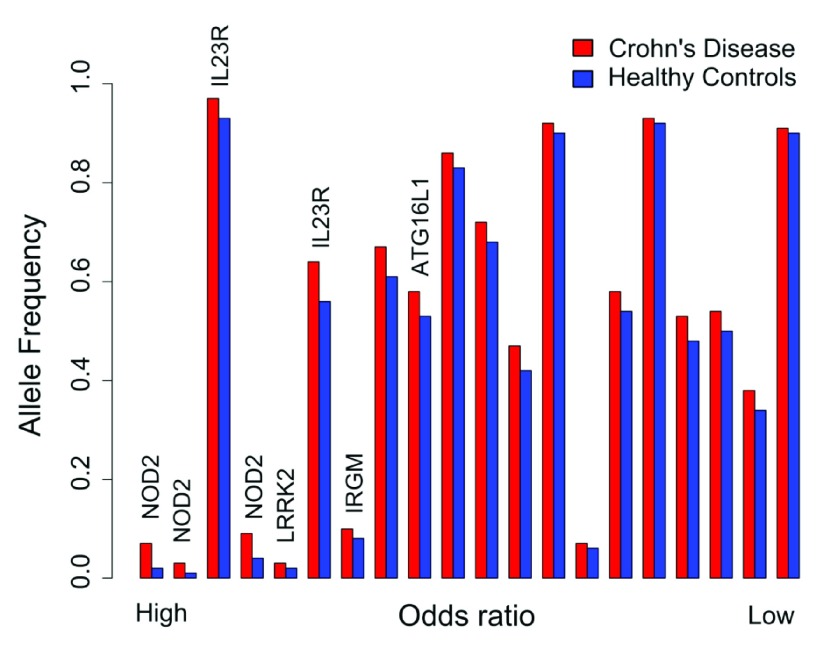
The allele frequency in Crohn’s disease patients and healthy controls for top 20 of 144 CD associated GWAS SNPs sorted by odds ratio. The data were taken from the European cohort in 276. Loci harbouring genes of interest have been indicated.

An important consideration is that about half of the healthy population also carry these variants, although (by definition) each at a slightly lower frequency than in the CD patients ( Figure 9). The healthy controls carrying these variants greatly outnumber the patients with CD in the population. With a prevalence of CD of about three patients in 1000 ^277^, and taking the NOD2 frameshift mutation as an example because it has the greatest effect size at 3.32, for every 100,000 individuals in the population there will be 99,680 unaffected individuals of whom ∼2390 will carry this mutation. In this population there will be ∼320 CD cases of which ∼48 will have the mutation ^274^. This means that the penetrance of this mutation, with by far the greatest association with the disease, is only 2%. These effect sizes pale into insignificance when compared with the effect size of HLA-B27 in ankylosing spondylitis of approximately 94 ^278, 279^, and HLA in type 1 diabetes and coeliac disease with effect sizes of approximately 25 and 50, respectively ^280^.

**Figure 10.**
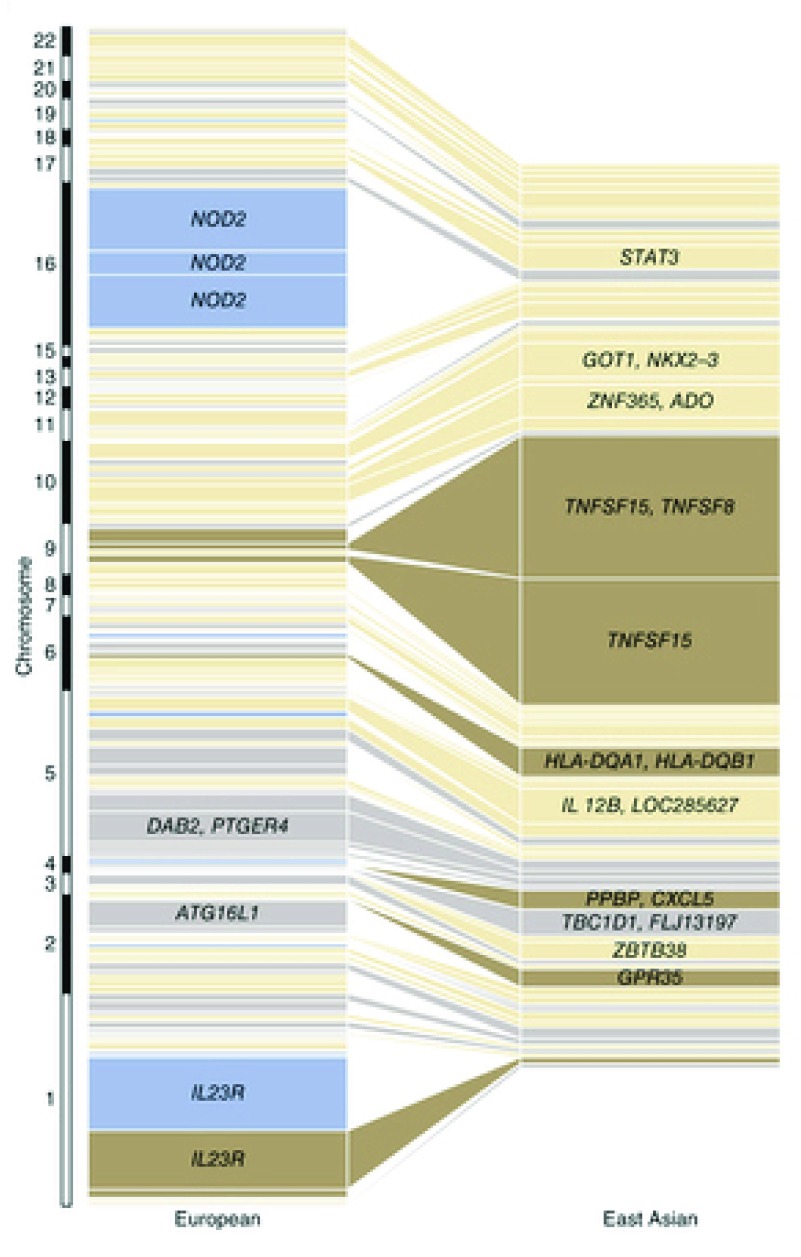
Comparison of the variance explained per risk variant for Crohn's disease between East Asians and Europeans. Each box represents an independently associated locus and the size of each box is proportional to the amount of variance in disease liability accounted for by that locus in the respective population. From 276 (reproduced with permission from the publisher).

GWAS conducted on Europeans and East Asian populations have yielded noticeably different findings ( Figure 10) ^276^. In East Asian populations, the loci with largest effect sizes were those harbouring the genes *TNFSF15/TNFSF8* (genes encoding cytokines that belong to the tumour necrosis factor (TNF) ligand family ^281^) and the major histocompatibility complex. Variants in *NOD2* and *ATG16L1* demonstrated no association with CD in these populations, and the effect size of the *IL23R* locus was minimal. The significant heterogeneity in common variant CD genetic architecture between different populations provides a further indication that the genes identified by GWAS are unlikely to play a primary causal role in the development of the disease, the manifestations of which are similar in patients regardless of their ethnicity.

GWAS have been performed for many different diseases and IBD associated loci have been shown to be shared with several other immunologically mediated diseases including rheumatoid arthritis, systemic lupus erythematosus (SLE), ankylosing spondylitis, coeliac disease and sarcoidosis ^282^. These associations are not surprising as comorbidities of some of these conditions are well recognised in the context of IBD ^283– 285^ and because almost all are associated with an increased incidence of similar pathologies such as arthritis ^286^, uveitis ^287, 288^ and bowel inflammation ^289– 292^.

Many of the associated genes common to these conditions have been implicated in pathways leading to activation or regulation of the immune response ^282^. It is possible that these genes were highlighted as being common to the chronic inflammatory conditions because they lead to more florid manifestations, causing signs and symptoms of the disease in those individuals with an underlying predisposition, thereby bringing them to the attention of the medical profession.

Despite their unimpressive effect sizes, the main GWAS CD associated molecules have generated considerable attention and are hence worth of a brief summary.

***NOD2.*** NOD (Nucleotide-binding Oligomerisation Domain) 2 is a member of an extended family of inflammatory and immune proteins in plants (the resistance (R) genes ^293^), Drosophila (Toll-like receptors ^294^) and animals (NOD families). These proteins combine a central nucleotide-binding domain (NOD) with a C-terminal leucine-rich repeat (LRR) motif and an N-terminal caspase recruitment domain (CARD) or equivalent.

In general these proteins recognise a signal from an invading organism in their leucine-rich domain (LRR) domain that induces a polymerisation that triggers a signalling cascade which terminates in the production and release of pro-inflammatory molecules. NOD2 is activated by muramyl dipeptide (MDP) a component of the cell wall of both Gram negative and Gram positive bacteria. It seems to be taken into the cells within endocytic vacuoles; presumably the organisms are then digested within this compartment and the solubilised MDP is moved into the cytoplasm by peptide transporters like *SLC15A3* ^295^. Very recently it has been demonstrated that NOD1 and NOD2, do not only respond to bacterial stimuli, but are also important mediators of ER-stress-induced inflammation (described in more detail below) ^296^.

In the resting state NOD2 is doubled back on itself in an auto-inhibited conformation in the cytoplasm until activated by the attachment of MDP upon which opens it allowing self-oligomerisation and the binding of ATP ^297, 298^. A series of phosphorylation steps then end in the translocation of NF-κB to the nucleus and the production of pro-inflammatory cytokines and antimicrobial peptides ^297^. This theoretical model of NOD2 function as a pattern recognition receptor capable of inducing pro-inflammatory cytokine secretion has been validated by *in vivo* studies in humans in which the application of MDP to skin windows induced the production and release of pro-inflammatory cytokines in healthy and CD patients without NOD2 mutations, but not in those carrying the CD-associated mutations ^216^. The impaired secretion of these inflammatory mediators into skin windows in the absence of MDP, in CD patients without mutations in NOD2 in this study, demonstrates that other pro-inflammatory signals and pathways must be abnormal in these subjects, indicating that there are at least two parallel routes initiating the inflammatory response.

Expression of NOD2 is largely restricted to peripheral blood monocytes ^299^ and to Paneth cells at the base of intestinal crypts ^300^. Monocytes constitute approximately 5% of the circulating leukocytes and are generally regarded as functioning predominantly as circulating precursors of macrophages, without much in the way of a distinct set of functions of their own. It seems intuitively unlikely that a highly specialised cell with an active NADPH oxidase and granules containing myeloperoxidase, all of which are lost with the transformation to macrophages, would be produced to act predominantly as a stem cell.

It is generally assumed that pro-inflammatory cytokines are secreted by tissue macrophages, but these are quite widely dispersed amongst tissues and could not accumulate as rapidly as needed at inflammatory sites as monocytes that are carried there in capillaries perfusing the region ^301, 302^. Monocytes are rapidly recruited to sites of acute inflammation where they extravasate into the tissues ^303^ and make a large contribution to the production of pro-inflammatory cytokines ^304– 306^ before being transformed into inflammatory macrophages.

***Autophagy and ATG16L1, CALCOCO2/NDP52, LRRK2 and Optineurin.*** First described in the new-born mouse kidney by Clark in 1957 ^307^ and reviewed by De Duve and Wattiaux ^308^, autophagy was initially described as a process in the cytoplasm of cells directed to the remodelling of tissues and the removal or damaged or effete organelles and proteins, and to partial self-digestion under starvation conditions.

To undertake this process, cells must first identify the region of cytoplasm, effete organelles, or invading microbes, as objects for engulfment. This is achieved by labelling the surface of the target with chains of a small protein, ubiquitin ^309, 310^. The ubiquitinated material is then encircled by a double membranous structure, produced from elongated vesicles at the Golgi apparatus or (ER) ^311^, the ends of which then fuse to form the characteristic vacuole with a double membrane. This autophagocytic vacuole then fuses with lysosomes containing enzymes that digest the inner membrane and its contents ^312^.

Membrane vesicle extrusion, ubiquitination and fusion of granules with vesicles are general biological processes that are involved in many diverse cellular functions in addition to autophagy. Most of the cellular machinery required for these processes in autophagy was identified in mutant *Saccharomyces cerevisiae* ^313^ and homologues were then then found in *Drosophila* and man. The molecular basis of human autophagy has largely been investigated in promyelocytic HL60 and the Human Embryonic Kidney (HEK293) cell lines ^314, 315^. Thus the autophagy molecules have been identified in assays that measure autophagy in primitive cells. This does not mean that these proteins are necessarily exerting their effects exclusively through autophagy in more mature cells and tissues.

What is probably of particular relevance to CD is the specialised type of autophagy, known as xenophagy, that is designed to deal with bacteria that escape into the host cytosol (such as *Shigella* or *Listeria*) or those that reside in a modified intracellular vacuole (such as *Salmonella* and *Mycobacteria*), which is important for the entrapment and lysosome-mediated degradation of bacterial pathogens ^316– 318^. CD resulting from abnormal xenophagy is seen in Niemann-Pick disease type C1 and XIAP deficiency with NOD2 variants in Crohn's disease ^319^. Other proteins implicated in xenophagy are the autophagy receptor CALCOCO2/NDP52 ^320^, Leucine-rich repeat kinase 2 (LRRK2) ^321^ and Optineurin ^322, 323^.

Despite the current interest in autophagy and in exploring the removal of intracytoplasmic organisms from intestinal cells and macrophages by xenophagy, it must not be forgotten that the vast majority of bacteria entering the body are phagocytosed, killed and digested by neutrophils, and that xenophagy only has to deal with a tiny minority escaping this process.

***ATG (autophagy-related)16L1.*** Attention was focussed upon ATG16L1 when an association was demonstrated with CD in a GWAS study ^324^. The SNP was a common, non-synonymous variant resulting in an amino acid change from threonine to alanine at position 300 of the full-length protein (T300A) with an odds ratio of only 1.26 ^274^. The ATG16L1 protein was shown to be mainly expressed in the thymus, prostate, liver, kidney and colon with little in the small bowel, and equal levels were found in the bowel from healthy and CD subjects with and without the polymorphism.

ATG16L1 is recruited to the ER at the initiation of the formation of the autophagosome at this site ^315^. It is involved in binding to ubiquitinated cytosolic *Salmonella* whilst these are enveloped in the phagophore to form the autophagosome ^325^ and could be important for the clearance of bacteria from within cells ^315, 326– 328^. This protein could be related to CD through the impaired clearance of intracellular organisms. It has been reported that the cellular architecture of Paneth cells is grossly distorted in knock-out mice severely depleted in this protein ^329^ and in CD patients homozygous for the T300A polymorphism. In the mice, there were many fewer granules in these cells, with their normal contents lying predominantly in the cytoplasm, together with multiple large vesicular structures that might represent the membranes that would normally surround granules. The rest of the bowel looked normal ^329^. It was also reported that CD patients with the T300A polymorphism demonstrated similar morphological changes with disorganized, or diminished, granules or exhibiting diffuse cytoplasmic lysozyme staining. This was claimed to be the first indication that Atg16L1 has a specific role in humans and mice in regulating the specialized properties of Paneth cells, and provides a novel and relevant mouse model that emulates one of the many diverse pathological hallmarks of human CD. Unfortunately the morphology of Paneth cells was not examined in healthy humans with the same polymorphism, which would not have been difficult given that 27% of this population is homozygous for the “risk” allele and 50% heterozygous, and which makes a pathological role for this SNP highly improbable ^274^.

It has been reported that the T300A significantly increases ATG16L1 sensitisation to caspase-3-mediated processing ^330^. Metabolic stress in human and murine macrophages increased degradation of the T300A (or mouse equivalent) variants of ATG16L1, respectively, resulting in diminished autophagy and knock-in mice harbouring the variant showed defective clearance of *Yersinia enterocolitica* and an elevated inflammatory cytokine response. Deletion of the caspase-3-encoding gene or elimination of the caspase cleavage site by site-directed mutagenesis rescued starvation-induced autophagy and pathogen clearance.

***IRGM***. Immunity related p47 guanosine triphosphatases (GTPases) or IRGs are a group of 47–48 kDa proteins that are implicated in the eradication of organisms like mycobacteria that are taken into cells within phagocytic vacuoles. IRGM has been characterised as an autophagy related molecule ^331^ on the basis that it was associated with vacuoles thought to contain mitochondria, a characteristic in common with autophagocytic vacuole. The evidence for the mitochondrial contents was the accumulation in the vacuoles of Mitotracker Red, a dye that partitions in mitochondria by virtue of the charge across their membranes. Mitochondria are marked for degradation by ubiquitination when they have lost their membrane potential ^332^ and this situation would not be improved by degradation within an autophagosome, so it is unlikely that such structures would attract Mitotracker Red. On the other hand, potentials are developed across the membrane of the phagocytic vacuole by the NADPH oxidase ^333^ and by electrogenic proton pumping of the vacuolar V-ATPases ^334^. The classification of IRGM as primarily involved in the autophagocytic processes on the evidence provided for mitophagy should be reconsidered.

The IRGs were discovered in mice as interferon-gamma inducible elements that were important for resistance to microbes engulfed into phagocytic vacuoles. There are 23 of these molecules in mice but only one or very few in humans ^335^. In human cells IRGM is largely expressed in immune cells whereas in mice it is almost exclusively in macrophages in which its expression is massively increased by LPS ( ^336^GeneAtlas MOE430 gcrma, Probeset: 1418825_at). Phagosomal maturation ^337, 338^ occurs through sequential fusion of the phagosome with early endosomes and lysosomes, resulting in acidification of the phagosome, release of hydrolytic proteases and death and digestion of the phagosome-bound pathogen. IRGs are located in the ER or Golgi compartments and after the host cell is infected, they are transported to pathogen-containing phagosomes/vacuoles, where they modulate the formation or processing of the phagosome, undermining pathogen survival either directly or by facilitating the action of intracellular effector molecules.

***XBP1 (X-box binding protein 1) and ER stress, or unfolded protein response (UPR)*** ^339, 340^. The ER is the principal organelle involved in the synthesis, maturation, and post- or co-translational modification of secreted and membrane proteins, as well as in various metabolic processes including dynamic ion storage and biogenesis of membrane structures ^341^. Properly folded proteins are then directed to the Golgi apparatus, to other intracellular organelles, and to the extracellular surface by the secretory pathway ^342^. The ER stress or unfolded protein response develops when the protein synthetic process becomes disordered; leading to the accumulation of dysfunctional unfolded protein as a result of genetic or adverse environmental factors. To deal with this, the unfolded protein triggers the unfolded protein response (UPR) ^343^ which includes reducing protein synthesis by downregulating mRNA translation, synthesising more molecular chaperones to assist protein folding, and degrading misfolded proteins which are ubiquinated and directed to the proteasomes. XBP1 is a transcription factor that plays a central role in activating these ER stress responses ^344^, and unexpectedly, NOD1 and NOD2 have been shown to be important mediators of this process ^296^. Abnormalities in the ER stress response will produce unfolded proteins that will require removal by autophagy, so the linkage between these two processes is to be expected ^345^.

Major demands are made on the protein synthetic machinery in rapidly turning over cells like the intestinal mucosa, and this is particularly true of cells like Paneth and goblet cells that secrete proteins in addition to attending to their own homeostatic requirements. When additional requirements for protein synthesis are called upon, induced, for example, by infection or chemical toxins, defects in the ER stress response could weaken the mucosa leading to ulceration ^346^.

***LRRK2***. A considerable amount of work has been done on this protein because mutations in LRRK2 cause familial and sporadic Parkinson’s disease (PD). It is a large, 280kDa, protein with GTPase and kinase domains, the latter being constitutively active in PD. It is found in immune cells, in lamina propria macrophages, B-lymphocytes and dendritic cells, and levels are markedly increased in the bowel in CD ^321^ and in microglia in the nervous system ^347^. It interacts with the small GTPases Rab32 and Rab38 with which it co-locates to transport vesicles and recycling endosomes ^348^ and it is important for the elimination or intracellular *Salmonellae* ^349^ and *Legionella* ^350^. Rab32 and Rab38 play an important role in the biogenesis and traffic of melanosomes and lysosomes and this system is disordered in Hermansky-Pudlak syndrome ^351^, accounting for the characteristic partial albinism. If LRRK2 and its associated proteins are important for immunological resistance to the development of CD then it might be expected that CD would be more common in conditions in which the LRRK2 system is disordered, which is in fact the case. A clear association exists between CD and PD ^352, 353^ and CD and Hermansky-Pudlak ^354^. At least two recent studies have discovered a link between mutations in LRRK2 and CD in Ashkenazi Jews (submitted for publication).

## Summary of outcome of GWAS studies in CD

The GWAS studies have provided a series of clear answers. No single gene, or a small number of genes, has been identified that is causal for CD. More than 170 GWAS hits combined contribute to about 10% of the “heritability” of CD. With the average individual contribution of only 0.1% it is unlikely that these variants will individually have major effects on cellular function, either in the CD patients or in experimental systems.

One of the founding premises of GWAS was that it was designed to identify genes that are involved in pathways relevant to disease pathogenesis. The value of this data, therefore, is that it points towards the specific pathways that are likely to be involved in disease pathogenesis, rather than identifying causal genes. Collectively, genes associated with CD by GWAS are enriched for Gene Ontology (GO) annotations (statements describing the functions of specific genes) related to host-microbe interactions, the regulation of cytokine production, lymphocyte activation and the response to molecules of bacterial origin ^273^. This is in keeping with the large body of data that is accumulating as to the role of the innate immune system and its interaction with intestinal microbes in the causal mechanisms in CD.

## Other investigations to identify causal molecules

### Macrophage expression profiling

In the knowledge that the release of pro-inflammatory cytokines by macrophages from CD subjects is depressed as a result of impaired vesicle trafficking ^219^, an attempt was made to identify genes contributing to this deficiency by looking for outlier levels of gene expression in these cells. The most commonly under-expressed gene identified was Optineurin, low expression levels of which were found in 10% of CD patients studied ^355^. ADAMDEC1 was under-expressed in about 7% of patients.

Optineurin (Optn) ^322, 323^ is a 67 kDa protein, ubiquitously expressed, and its expression can be induced by TNFα and interferons, probably as a result of NFκB activation, and it is localised in the cytosol and Golgi apparatus. In essence it is a linker, or adaptor, molecule and has several binding partners including Rab8, Huntingtin, the gene for which is mutated in Huntington disease, and Myosin VI, a multifunctional motor protein. Rab8 is a small GTPase involved in vesicular trafficking between the trans-Golgi network (TGN) and the plasma membrane. The function of Huntingtin itself is unknown but it is associated with several factors involved in vesicle trafficking. Myosin VI is attached by OPTN to the Golgi apparatus and then it participates in the transport of vesicles and their protein cargos from the from the Trans-Golgi network to be released at the cell surface. OPTN also contains a ubiquitin binding domain with the ability to bind polyubiquitinated cargoes and transport them to autophagosomes via its microtubule-associated protein 1 light chain 3-interacting domain ^356^.

Macrophages from patients with low expression of OPTN secreted abnormally low levels of pro-inflammatory cytokines as do macrophages from OPTN knock out mice ^357^. mRNA expression levels of these cytokines were normal, consistent with deranged secretion rather than synthesis. These mice were more susceptible to infection with *Citrobacter, E. coli* and *Salmonella* ^358^, and showed reduced levels of TNFα in their serum, diminished neutrophil recruitment to sites of acute inflammation and greater mortality, than wild-type mice. OPTN-knockdown zebrafish infected with *Salmonella* also had a higher mortality ^357^.

ADAMDEC1 (ADAM-like Decysin-1) is a member of the ADAM (A Disintegrin And Metalloproteinase) family, the expression of which is restricted to the macrophage/dendritic cell populations of the gastrointestinal tract. Its biological function is unknown but it has been hypothesised to play a role in immunity. Reduced ADAMDEC1 expression in macrophages from a subgroup of CD patients has provided evidence of a potential role in bowel inflammation ^355^. Adamdec1 ^-/-^ mice were more susceptible to the induction of bacterial and chemical induced colitis and they cleared *Citrobacter rodentium* less efficiently than wild-type mice after infection ^359^.

### DNA sequencing

The development, availability and falling costs of high throughput DNA sequencing, has provided the means of directly identifying causal mutations in human disease ^360, 361^. Several studies employing such technology have been undertaken in CD ^362, 363^ and many more are likely to appear over the ensuing years. High-throughput DNA sequencing has also been absolutely crucial for the diagnosis of the rare primary immunodeficiencies ^47^ that produce bowel inflammation, rather than CD, as described earlier.

Initially the DNA of GWAS associated loci was sequenced, and a small number of rare variants were identified, most notably in the genes *CARD9* and *IL23R* ^364, 365^.

The major problem in identifying causal genes by sequencing is the considerable individual variation in DNA sequence. Asymptomatic individuals carry, on average, approximately 100 genuine loss of function variants with ∼20 genes completely inactivated ^366^. This makes it very difficult to identify the disease causing mutation/s in any one individual.

Of note, an ongoing study in which whole-genome sequencing has been undertaken in 2,697 CD cases and 3,652 healthy controls failed to identify a single variant at genome-wide significance that had not already been identified by GWAS ^367^.

Alternative approaches have been taken to overcome the difficulty of the interpretation of individual variation. Several studies have focussed on the analysis of Ashkenazi Jews (AJ) because they have a roughly fourfold increased incidence of CD and demonstrate genetic homogeneity, having arisen from approximately 350 individuals about 30 generations ago ^368^.

Chuang *et al.* sequenced the exomes of 50 AJ CD patients and prioritised low frequency coding variants which were then genotyped in approximately 3,000 AJ CD cases and 3,000 controls. They identified a frameshift mutation in *CSF2RB* as a strong causal candidate which was associated with CD at p<3.5×10 ^-6^ and an OR of 1.5 ^369^. This variant is rare in the non-AJ population.

Levine *et al*. utilised an alternative family based approach ^370^. They characterised two very large AJ families with >800 and >200 members with 54 and 26 affected cases respectively, sequenced the exomes of all cases and imputed the genotypes of the unaffected family members. Low frequency coding variants that were predicted to be damaging and were enriched in affected compared with unaffected individuals were prioritised. In the large family they independently identified the identical frameshift mutation in CSF2RB as concurrently reported by Chuang *et al*. as a likely causal variant. Other strong candidate genes included *NLRP2*, a NOD-like receptor and a component of the inflammasome; *ZC3H18* which is involved in IKK and NFκB activation ^371^, a pathway of established importance in CD; and *MEGF10,* a phagocytic receptor involved in apoptosis ^372^.

### CSF2RB

Having been identified independently by two groups *CSF2RB* must be considered as a causal gene for CD in AJs ^373^. CSF2RB is the common or shared β subunit of the receptors for granulocyte-macrophage colony-stimulating factor (GM-CSF), interleukin (IL)-3, and IL-5 ^374^. The distinct α chains of these receptors provide cytokine specificity whilst the β chain is responsible for high-affinity binding and is the major downstream signalling component of the receptor complexes.

GM-CSF is produced by myeloid cells, dendritic cells (DCs), T cells, B cells, and several non-immunological cells including epithelial cells ^375^ following exposure to inflammatory stimuli to promote the production and function of myeloid haemopoietic cells including haemopoietic progenitor cells and differentiated cells such as basophils, neutrophils, eosinophils, macrophages and certain dendritic cells ^376^ to deal with the cause of the inflammation.

IL-3 is predominantly produced by activated T cells, natural killer (NK) cells and mast cells. It acts on the early stages of haematopoiesis in synergy with other cytokines to induce progenitors of various lineages but it is a very important stimulus for the generation of mast cells and the regulation of mast cell function as well as basophil production and activation.

IL-5 stimulates mainly the production and function of eosinophils. The major source of IL-5 is T-cells with relatively lower amounts produced by mast cells and eosinophils ^377^.

### DUOX2

Levine *et al.* also identified a damaging missense mutation in *DUOX2* that impaired the function of the protein and showed a possible epistatic interaction with NOD2 ^370^. DUOX2 is a member of the large NADPH oxidase (NOX) family of enzymes ^378^. Its expression in the bowel epithelium is induced by the microbiota ^379^. It generates H _2_O _2_ at the mucosal surface and this acts as substrate for lactoperoxidase catalysed oxidation of thiocyanate to microbicidal hypothiocyanite ^6^. It might also attract neutrophils to inflammatory sites ^380^. Knockdown of the DUOX2 homologue in invertebrates and mice resulted in an impaired tolerance to enteric bacteria ^381^. Of relevance, given the possible NOD2 epistatic interaction observed, a physical and functional interaction between these proteins has been demonstrated in epithelial and HEK293 cells ^382^.

## Future treatment options

Treatment of CD poses a conundrum. The logical approach to correcting the underlying problem would be to develop means of enhancing innate immunity, although at present no such range of drugs is currently available. It would be dangerous to attempt to do this in the presence of ongoing bowel inflammation but it could be useful to maintain patients in remission after they had been cleared of disease by surgical resection, or through the use non-immunosuppressant therapies such as elemental diets ^383^. This approach was attempted with levamisole ^384^ as the immunostimulant, with varying results ^385, 386^. One problem with this form of treatment is that it is important to ensure that the patients are in remission before it is commenced; otherwise it is likely to exacerbate the inflammation. In two studies levamisole induced a severe reversible polyarthropathy ^387, 388^, indicating that the drug was in fact altering the immunological/inflammatory axis, and providing clues as to IBD associated arthritis, and to the immunopathology of the idiopathic arthritides in general.

Current drug and biological treatments are largely immunosuppressant. This, to varying degrees of efficiency, dampens down the secondary inflammation induced by the retained foreign material within the tissues. Anti-TNF treatments can be very helpful but do not provide a comprehensive answer. Only one third of patients will be in remission after one year on these treatments ^389^. Immunosuppressant treatment further compromises the underlying innate immune deficit to mucosal damage, thereby increasing the likelihood of further infection and the influx of bowel contents into the tissues, possibly converting CD from a sporadic to a chronic condition.

The primary pathology in most case of CD appears to affect macrophages recruited from the blood as monocytes ^301^. Advances in gene editing with the CRISPR-Cas ^390, 391^ technology make the corrective treatment of CD a real possibility in the relatively near future. Once a primary causal mutation has been identified, and validated in animal models, bone marrow could be extracted, edited and reinfused into a conditioned patient in much the same way as is being applied to gene therapy for primary immunodeficiencies ^392^.

## Conclusion

After almost a century of concerted effort in clinical investigations combined with recent technological developments in genomic medicine, a consensus view is developing as to the causes of CD.

A genetic predisposition to the condition can exist, as evidenced by twin and family studies. Studies in patients have clearly demonstrated that there is a defect in the acute inflammatory response resulting in an impaired recruitment of neutrophils to inflammatory sites and a consequent delay in the clearance of bacteria from them. This results from an initial blunted response by monocytes and macrophages leading to deficient secretion of pro-inflammatory cytokines.

CD is a syndrome and the molecular basis of this deficiency will vary considerably. It might reflect the multifactorial extreme end of a normal distribution, or the critical loss of one or more important molecules. Molecules contributing to the former will be very difficult to identify whereas some of those playing a more singular role have been highlighted by linkage, GWAS and DNA sequencing studies. These extend across the spectrum of the interface between microbes and cells: from the derangement of cellular homeostasis, probably in intestinal and immune cells, through impairment of the ER stress response (XBP1 malfunction); defective signalling (aberrant NOD2); depressed killing and digestion of organisms within phagocytic vacuoles (IRGM) or xenophagic recovery of those that escape into the cytoplasm (ATG16L1); failure of vesicle trafficking resulting in disorganised lysosomal biology of reduced cytokine secretion (LRRK2, Optineurin) or ineffective signalling of cytokines on target cells because of aberrant receptors (CSF2RB). In addition, primary abnormalities of neutrophils, the final effector cells, constitute the ultimate predisposition.

However severe the covert underlying predisposition, it requires a triggering factor to be expressed, and all the evidence points to this being an enteric infection. The identity of the organism is of secondary importance to the fact that the mucosa is breached, allowing ingress of the faecal contents into the bowel wall. Increased frequency or potency of these infections, as a result of factors implicated in the “Hygiene Hypothesis” or through increased spread by travel, or changes in sexual practises, could account for the increasing incidence of CD in the Western world.

It is the abnormal response to the penetrating faeces that is the common denominator of the CD syndrome, and it is the failure to eliminate the foreign material in the tissues that leads to the classical chronic granulomatous inflammation, and subsequently to an adaptive immune response. The counter intuitive, but logical, conclusion is that a disease characterised by grossly exuberant inflammation can result from an initial failure of innate inflammation. A similar mechanism might be responsible for other chronic inflammatory conditions of unknown aetiology, like for example sarcoidosis, ankylosing spondylitis, psoriasis, rheumatoid arthritis and SLE.
