# Supplementary material for: Making sense of the cause of Crohn’s – a new look at an old disease
Source: F1000Res. 2016 Nov 16;5:2510. Originally published 2016 Oct 12. [Version 2] doi: 10.12688/f1000research.9699.2 (PMC5224693; doi:10.12688/f1000research.9699.2)
Supplement: Peer review history

# Peer review: Referee response for version 2

**DOI:** 10.5256/f1000research.10828.r17726

**Published:** 2016-11-21

**Authors:** James C. Lee

**Affiliations:** 1 Department of Medicine, Cambridge Institute for Medical Research, University of Cambridge, Cambridge, UK

I think the revisions are helpful and agree that GWAS has not identified single genes that can cause Crohn's disease. The revised text highlights both this point and that the value of GWAS lies in identifying pathways that may be involved. So as not to dissuade investigators from studying the effects of disease-associated genetic variants, however, I think it is important to note that a small odds ratio does not mean that variants cannot have effects on cellular function (for reasons including functional redundancy and overlap as stated in my first review). For example, several GWAS hits have been shown to drive relevant *in vitro* and *in vivo* phenotypes, which - through their elucidation - have added to our understanding of disease:

 Nonetheless, I am happy to approve this manuscript, which I believe presents a thorough overview of the pathogenesis of Crohn's disease and is an important contribution to the literature.

I have read this submission. I believe that I have an appropriate level of expertise to confirm that it is of an acceptable scientific standard.

## References

[1] MusunuruKStrongAFrank-KamenetskyMLeeNEAhfeldtTSachsKVLiXLiHKuperwasserNRudaVMPirruccelloJPMuchmoreBProkunina-OlssonLHallJLSchadtEEMoralesCRLund-KatzSPhillipsMCWongJCantleyWRacieTEjebeKGOrho-MelanderMMelanderOKotelianskyVFitzgeraldKKraussRMCowanCAKathiresanSRaderDJ: From noncoding variant to phenotype via SORT1 at the 1p13 cholesterol locus. *Nature*.2010;466(7307) : 10.1038/nature09266 714-9 10.1038/nature09266 20686566PMC3062476

[2] LeeJCEspéliMAndersonCALintermanMAPocockJMWilliamsNJRobertsRViatteSFuBPeshuNHienTTPhuNHWesleyEEdwardsCAhmadTMansfieldJCGearryRDunstanSWilliamsTNBartonAVinuesaCGParkesMLyonsPASmithKG: Human SNP links differential outcomes in inflammatory and infectious disease to a FOXO3-regulated pathway. *Cell*.2013;155(1) : 10.1016/j.cell.2013.08.034 57-69 10.1016/j.cell.2013.08.034 24035192PMC3790457

[3] MurthyALiYPengIReicheltMKatakamAKNoubadeRRoose-GirmaMDeVossJDiehlLGrahamRRvan Lookeren CampagneM: A Crohn's disease variant in Atg16l1 enhances its degradation by caspase 3. *Nature*.2014;506(7489) : 10.1038/nature13044 456-62 10.1038/nature13044 24553140

[4] OldridgeDAWoodACWeichert-LeaheyNCrimminsISussmanRWinterCMcDanielLDDiamondMHartLSZhuSDurbinADAbrahamBJAndersLTianLZhangSWeiJSKhanJBramlettKRahmanNCapassoMIolasconAGerhardDSGuidry AuvilJMYoungRAHakonarsonHDiskinSJLookATMarisJM: Genetic predisposition to neuroblastoma mediated by a LMO1 super-enhancer polymorphism. *Nature*.2015;528(7582) : 10.1038/nature15540 418-21 10.1038/nature15540 26560027PMC4775078

# Peer review: Referee response for version 2

**DOI:** 10.5256/f1000research.10828.r17727

**Published:** 2016-11-16

**Authors:** Jean-Laurent Casanova

**Affiliations:** 1 St. Giles Laboratory of Human Genetics of Infectious Diseases, Rockefeller University, New York, NY, USA

I have read this submission. I believe that I have an appropriate level of expertise to confirm that it is of an acceptable scientific standard.

# Peer review: Referee response for version 1

**DOI:** 10.5256/f1000research.10455.r17018

**Published:** 2016-10-27

**Authors:** James C. Lee

**Affiliations:** 1 Department of Medicine, Cambridge Institute for Medical Research, University of Cambridge, Cambridge, UK

This review presents a thorough overview of the pathogenesis of Crohn's disease, drawing on a broad range of evidence, from epidemiology to human immunology to genetics. It is impressive to have collated such diverse topics into a single review, and therefore understandable that some areas are discussed in more detail than others. Overall this is a valuable contribution to the literature.

 Of note, some parts of this review read more like a perspective piece and draw conclusions that I suspect are not widely held, although they are supported by the evidence presented. There is also a considerable focus on the author's own work in several sections, although this is understandable and it is valuable to have these experiments discussed in the context of the broader literature regarding Crohn's disease pathogenesis. 

 The author is fairly dismissive of the role of genetics, and more specifically of the significance of disease-associated genetic variants, based on their small odds ratios and frequency within the healthy population. While I understand this opinion, I think it is important to clarify a couple of these points for a general audience. First, it is important to note that one of the founding premises of GWAS was that it was designed to identify genes that are involved in pathways relevant to disease pathogenesis. It was not expected - based on the common disease-common variant hypothesis - that any of the SNPs or genes would by themselves be causal. To take the example of impaired autophagy - something that has been commonly reported in Crohn's patients -  there are several ways in which this might occur. For instance some patients may carry NOD2 or IRGM or ATG16L1 mutations, while in others it may be due to environmental factors such as smoking - see Monick *et al*. (2010). Based on the multi-hit model the author proposes, the key point is that "hits" are simultaneously required in several pathways in conjunction with an environmental trigger in order for a disease to occur. Because the exact nature of each hit is likely to vary between patients, it is not surprising that univariate genetic analysis identifies small odds ratios at individual SNPs (many of which may have similar effects on a given pathway). Moreover, the size of these effects may be different when gene-environment and epistatic interactions are taken into account. The value of this data, therefore, is that it points towards the specific pathways that are likely to be involved in disease pathogenesis, rather than identifying causal genes.

 This is perhaps best illustrated in cardiovascular medicine, where genetic variants in the gene that encodes HMG-CoA reductase have been significantly associated with LDL cholesterol levels, but the odds ratios for these associations are very small (presumably because there are many other causes of elevated cholesterol, Kathiresan *et al.* (2008)). Despite this, pharamcological targeting of this enzyme with statins represents the most effective treatment for hypercholersterolaemia. As such, a small odds ratio at individual SNP should not be confused with the implicated gene or pathway not being biologically or therapeutically important. 

 Other points:

- Discussion of the ATG16L1 variant would benefit from including the recent evidence linking the variant to susceptibility to caspase-mediated degradation (Murthy *et al. *(2014)).
- It would be worth highlighting the cross-talk between ER stress and autophagy, particularly in the context of Crohn's (Adolph *et al.(*2013)). 
- p.2: IL-23 is thought to be more involved in the expansion and survival of Th17 than in transforming naive T cells into Th17 cells
- p.7 typo: Danziel should be Dalzeil

I have read this submission. I believe that I have an appropriate level of expertise to confirm that it is of an acceptable scientific standard, however I have significant reservations, as outlined above.

## References

[1] AdolphTETomczakMFNiederreiterLKoHJBöckJMartinez-NavesEGlickmanJNTschurtschenthalerMHartwigJHosomiSFlakMBCusickJLKohnoKIwawakiTBillmann-BornSRaineTBhartiRLuciusRKweonMNMarciniakSJChoiAHagenSJSchreiberSRosenstielPKaserABlumbergRS: Paneth cells as a site of origin for intestinal inflammation. *Nature*.2013;503(7475) : 10.1038/nature12599 272-6 10.1038/nature12599 24089213PMC3862182

[2] KathiresanSMelanderOAnevskiDGuiducciCBurttNPRoosCHirschhornJNBerglundGHedbladBGroopLAltshulerDMNewton-ChehCOrho-MelanderM: Polymorphisms associated with cholesterol and risk of cardiovascular events. *N Engl J Med*.2008;358(12) : 10.1056/NEJMoa0706728 1240-9 10.1056/NEJMoa0706728 18354102

[3] MonickMMPowersLSWaltersKLovanNZhangMGerkeAHansdottirSHunninghakeGW: Identification of an autophagy defect in smokers' alveolar macrophages. *J Immunol*.2010;185(9) : 10.4049/jimmunol.1001603 5425-35 10.4049/jimmunol.1001603 20921532PMC3057181

[4] MurthyALiYPengIReicheltMKatakamAKNoubadeRRoose-GirmaMDeVossJDiehlLGrahamRRvan Lookeren CampagneM: A Crohn's disease variant in Atg16l1 enhances its degradation by caspase 3. *Nature*.2014;506(7489) : 10.1038/nature13044 456-62 10.1038/nature13044 24553140

# Response

**Published:** 2016-10-31

**Authors:** Tony Segal

**Affiliations:** Medicine, UCL, UK

I am most grateful to Dr Lee for taking the time and trouble to thoroughly review my manuscript and for his comments.

 I would like to address the issue of the relationship between GWAS and the identification of causal genetic variants. It is simple to establish causality for dominant, highly penetrant genes like polycystic renal disease, polyposis coli and Huntington’s disease. However it is more difficult when penetrance is incomplete, when one is reliant upon the odds ratio of the frequency of the variants in affected as compared with unaffected individuals. In general very high odds ratios would strongly suggest causality. Genes with very high odds rations have been identified by GWAS. For example:

 Birdshot chorioretinopathy

https://www.ncbi.nlm.nih.gov/pubmed/24957906

 However, the most important consideration is not whether or not the design of GWAS was to identify causal genes, but what the common perception is, and in many cases it is the perception that GWAS do in fact generally identify causal genes:

 The genetic background of Inflammatory Bowel Disease: From correlation to causality.

Uniken Venema WT *et al.*

 https://www.ncbi.nlm.nih.gov/pubmed/?term=27785786

 Insights into kidney diseases from genome-wide association studies.

Wuttke M and Köttgen A

https://www.ncbi.nlm.nih.gov/pubmed/?term=27477491

 Genetics of complex traits: prediction of phenotype, identification of causal polymorphisms and genetic architecture.

Goddard ME *et al.*

 https://www.ncbi.nlm.nih.gov/pubmed/?term=27440663

 A large genome-wide association study of age-related macular degeneration highlights contributions of rare and common variants.

Fritsche LG *et al*.

 https://www.ncbi.nlm.nih.gov/pubmed/?term=26691988

 Combining GWAS and RNA-Seq Approaches for Detection of the Causal Mutation for Hereditary Junctional Epidermolysis Bullosa in Sheep.

Suárez-Vega A *et al.*

 https://www.ncbi.nlm.nih.gov/pubmed/?term=25955497

 It was therefore very important to state in the review that GWAS have not identified genes that cause Crohn's disease. 

 Under the **Summary of outcome of GWAS studies in CD **I have now stated:

 The GWAS studies have provided a series of clear answers. No single gene, or a small number of genes, has been identified that is causal for CD. More than 170 GWAS hits combined contribute to about 10% of the “heritability” of CD. With the average individual contribution of only 0.1% it is unlikely that these variants will individually have major effects on cellular function, either in the CD patients or in experimental systems.

 One of the founding premises of GWAS was that it was designed to identify genes that are involved in pathways relevant to disease pathogenesis. The value of this data, therefore, is that it points towards the specific pathways that are likely to be involved in disease pathogenesis, rather than identifying causal genes.

# Peer review: Referee response for version 1

**DOI:** 10.5256/f1000research.10455.r16956

**Published:** 2016-10-17

**Authors:** Jean-Laurent Casanova

**Affiliations:** 1 St. Giles Laboratory of Human Genetics of Infectious Diseases, Rockefeller University, New York, NY, USA

This is a very comprehensive review of the pathogenesis of Crohn's disease (CD). The paper thoroughly covers all relevant aspects, including epidemiology, microbiology, immunology, and genetics. In that regard, it is both holistic and reductionist -- a very rare combination nowadays. It is beautifully written and presents a fair account of the current state of knowledge. Importantly, it is not biased: it does not resemble any of the too many reviews on CD that provide more beliefs and opinions than facts. This is in my view the very best review ever written on CD. This is clearly a must-read for anyone interested in this condition.

I have read this submission. I believe that I have an appropriate level of expertise to confirm that it is of an acceptable scientific standard.
